# *Lactobacillus acidophilus* Metabolizes Dietary Plant Glucosides and Externalizes Their Bioactive Phytochemicals

**DOI:** 10.1128/mBio.01421-17

**Published:** 2017-11-21

**Authors:** Mia C. Theilmann, Yong Jun Goh, Kristian Fog Nielsen, Todd R. Klaenhammer, Rodolphe Barrangou, Maher Abou Hachem

**Affiliations:** a Department of Biotechnology and Biomedicine, Technical University of Denmark, Kongens Lyngby, Denmark; b Department of Food, Bioprocessing and Nutrition Sciences, North Carolina State University, Raleigh, North Carolina, USA; University of Michigan; University of Hawaii at Manoa

**Keywords:** *Lactobacillus*, beta-glucoside, bioavailability, gut microbiota, phytochemical, polydatin, polyphenols, resveratrol, xenobiotic metabolism

## Abstract

Therapeutically active glycosylated phytochemicals are ubiquitous in the human diet. The human gut microbiota (HGM) modulates the bioactivities of these compounds, which consequently affect host physiology and microbiota composition. Despite a significant impact on human health, the key players and the underpinning mechanisms of this interplay remain uncharacterized. Here, we demonstrate the growth of *Lactobacillus acidophilus* on mono- and diglucosyl dietary plant glycosides (PGs) possessing small aromatic aglycones. Transcriptional analysis revealed the upregulation of host interaction genes and identified two loci that encode phosphotransferase system (PTS) transporters and phospho-β-glucosidases, which mediate the uptake and deglucosylation of these compounds, respectively. Inactivating these transport and hydrolysis genes abolished or severely reduced growth on PG, establishing the specificity of the loci to distinct groups of PGs. Following intracellular deglucosylation, the aglycones of PGs are externalized, rendering them available for absorption by the host or for further modification by other microbiota taxa. The PG utilization loci are conserved in *L. acidophilus* and closely related lactobacilli, in correlation with versatile growth on these compounds. Growth on the tested PG appeared more common among human gut lactobacilli than among counterparts from other ecologic niches. The PGs that supported the growth of *L. acidophilus* were utilized poorly or not at all by other common HGM strains, underscoring the metabolic specialization of *L. acidophilus*. These findings highlight the role of human gut *L. acidophilus* and select lactobacilli in the bioconversion of glycoconjugated phytochemicals, which is likely to have an important impact on the HGM and human host.

## IMPORTANCE

Thousands of therapeutically active plant-derived compounds are widely present in berries, fruits, nuts, and beverages like tea and wine. The bioactivity and bioavailability of these compounds, which are typically glycosylated, are altered by microbial bioconversions in the human gut. Remarkably, little is known about the bioconversion of PGs by the gut microbial community, despite the significance of this metabolic facet to human health. Our work provides the first molecular insights into the metabolic routes of diet relevant and therapeutically active PGs by *Lactobacillus acidophilus* and related human gut lactobacilli. This taxonomic group is adept at metabolizing the glucoside moieties of select PG and externalizes their aglycones. The study highlights an important role of lactobacilli in the bioconversion of dietary PG and presents a framework from which to derive molecular insights into their metabolism by members of the human gut microbiota.

## INTRODUCTION

The human gut microbiota (HGM) exerts a profound impact on human health and developmental biology (1–3), in part through interplay with diet as well as metabolism of xenobiotics (4) and nondigestible carbohydrates (5). These impressive metabolic capabilities are encoded by a vast metagenome that outnumbers the genes of the human genome by more than 150-fold. Preferential carbohydrate metabolism is a key factor that shapes the HGM (6–8), whereby specific taxa adapt to different biogeographic and metabolic niches in the gut (9–11). The impact of the HGM composition on health has been well established through metagenomics and association studies (2, 12), but the functional understanding of the interplay between HGM and various dietary components remains limited.

A plethora of phytochemicals occur in fruits, berries, nuts, and vegetables and also in beverages, such as wine and tea (13). These compounds are frequently glycoconjugated *in planta* to enable storage and solubility or to modulate biological activity (14). Several phytochemicals, e.g., some phenolic and polyphenolic compounds, exhibit beneficial health effects via anti-inflammatory, antiestrogenic, cardioprotective, anticarcinogenic, chemopreventative, neuroprotective, antimicrobial, or antioxidant properties (15, 16). The biological activity varies depending on the glycoconjugation of the phytochemical (17). Stimulation or lack of inhibition of growth of lactobacilli on a few available glycosylated phytochemicals, here referred to as plant glycosides (PGs), has been reported (18, 19), but the role of lactobacilli in the bioconversion of PG has not been clear to date.

*Lactobacillus acidophilus* NCFM, a widely used probiotic, is a well-characterized model for human gut-adapted lactobacilli (20, 21), owing to its tolerance to bile (22), adhesion to epithelial cells and mucus (23, 24), and ability to colonize the host. Additionally, the abilities of this strain to take up and catabolize a variety of nondigestible complex carbohydrates have been documented and implicated in gut persistence (25–27). Lactobacilli rely on phosphotransferase systems (PTS) in the uptake of most carbohydrates (28). The genomes of human gut lactobacilli are expanded with PTS genes, compared to genomes of counterparts from more carbohydrate-poor ecologic niches (e.g., dairy or food), but functional assignment is lacking for most of these transporters. To a lesser extent, lactobacilli also possess ATP-binding cassette (ABC) importers for uptake of nutrients and ABC exporters, for which the specificities of most are unknown.

Here, we demonstrate the growth of *L. acidophilus* NCFM on chemically diverse and nutritionally relevant PG. We also elucidate a new metabolic strategy, involving the uptake of PG via two different PTS and intracellular hydrolysis by specialized phospho-β-glucosidases (P-Bgls), followed by the externalization of the aglycone moieties into culture supernatants. The PG utilization loci are conserved in the *L. acidophilus* species and closely related lactobacilli and correlate with versatile growth on these compounds. Growth on the tested PG appeared more common in human gut lactobacilli than for counterparts from other ecologic niches. The PG that supported the growth of *L. acidophilus* were utilized poorly or not at all by other common HGM strains, underscoring the metabolic specialization of *L. acidophilus*. These findings highlight the role of human gut *L. acidophilus* and select lactobacilli in modulating the availability and bioactivity of glycoconjugated phytochemicals, and these modulations are likely to have an important impact on the HGM and their host.

## RESULTS

### *L. acidophilus* NCFM grows on nutritionally relevant plant glycosides.

**FIG 1:**
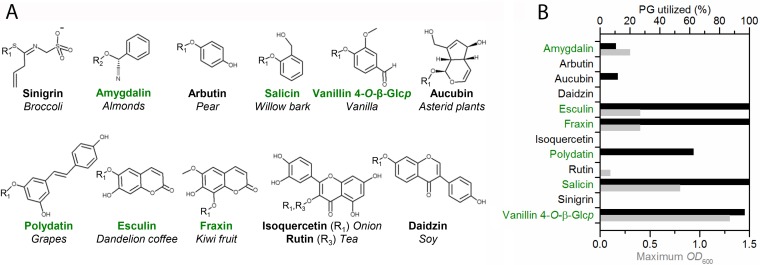
Growth of *Lactobacillus acidophilus* NCFM on plant glycosides. (A) Structures and common sources of plant glycoside substrates in this study. The compounds that support growth of *L. acidophilus* are shown in green. R1, β-d-Glc*p*; R2, gentiobioside [β-d-Glc*p*-(1,6)-d-Glc*p*]; R3, rutinoside [α-l-Rha*f*-(1,6)-d-Glc*p*]. (B) Graph showing results of PG utilization mass spectrometry analysis (black bars) and growth (gray bars, maximum OD_600_) after 24 h of at least biological triplicates. Standard deviations were <13% of the maximum OD_600_ for PGs that sustained growth. Due to the low solubility of polydatin, the OD_600_ could not be used as a growth metric and utilization of this compound was confirmed by the production of lactate as well as a high utilization level based on the metabolite analysis.

The growth of *L. acidophilus* NCFM was evaluated on 11 chemically diverse, nutritionally relevant, and/or therapeutically active PG after 24 h of growth (Fig. 1; see Table S1 in the supplemental material). The cyanogenic diglucoside amygdalin, coumarin glucosides esculin and fraxin, alcoholic glucoside salicin, and aldehyde glucoside vanillin 4-*O*-β-glucoside all supported growth to a maximum optical density at 600 nm (OD_600_) of 0.3 to 1.3 in 200-µl cultures in 96-well plates. The poor solubility of the stilbenoid polydatin precluded use of the OD_600_ as a growth metric, but growth on this bioactive compound was verified by the production of lactate and metabolite analysis (described below). Additional *Lactobacillus* strains from different ecologic niches were tested for growth on the PGs amygdalin, arbutin, esculin, and salicin, as well as the control disaccharides, cellobiose, and glucose. *L. acidophilus* displayed versatile growth on PG, together with *Lactobacillus plantarum* subsp. *plantarum* and a *Lactobacillus rhamnosus* strain (Table S2). Generally, the ability to grow on PG was more common in strains isolated from the human gut niche than in counterparts from other ecological environments.

10.1128/mBio.01421-17.3 TABLE S1  Plant glycosides studied in this work and their ability to support growth (OD_600_) of *L. acidophilus* NCFM. Download TABLE S1, DOCX file, 0.03 MB. Copyright © 2017 Theilmann et al. 2017 Theilmann et al. This content is distributed under the terms of the Creative Commons Attribution 4.0 International license.

10.1128/mBio.01421-17.4 TABLE S2  Growth of *Lactobacillus* species on selected plant glucosides, cellobiose, or glucose. Values for growth reflect corrections to the growth level in medium without carbohydrate. +++, OD_600_ max > 0.6; ++, 0.6 > OD_600_ max > 0.3; +, 0.3 > OD_600_ max > 0.1; −, OD_600_ max < 0.1. Download TABLE S2, DOCX file, 0.02 MB. Copyright © 2017 Theilmann et al. 2017 Theilmann et al. This content is distributed under the terms of the Creative Commons Attribution 4.0 International license.

### Growth on plant glycosides upregulates carbohydrate metabolism and host interaction genes in *L. acidophilus* NCFM.

Global transcription was analyzed by transcriptome sequencing (RNA-Seq) in early- to mid-exponential-phase *L. acidophilus* NCFM cultures growing on lactose or glucose, as well as the growth-supporting PGs amygdalin, esculin, and salicin, which were selected based on their availability and chemical diversity.

The growth on lactose and the PGs differentially upregulated less than 10% of the 1,832 predicted protein-coding genes, compared to glucose (Table S3). Only 2% of the genes were highly upregulated on PG (Table 1). Of the upregulated genes, 55 were shared by two or more of the PG, whereas 58, 35, and 0 were uniquely induced by amygdalin, esculin, and salicin, respectively, indicating more extensive and unique cellular responses to amygdalin and to a lesser extent esculin than to salicin. Amygdalin, which supported the lowest level of growth, interestingly upregulated the highest number of genes (116 genes), followed by esculin (87 genes) and salicin (33 genes).

10.1128/mBio.01421-17.5 TABLE S3  Differentially upregulated genes of *L. acidophilus* NCFM grown on different carbon sources. Carbon sources included amygdalin (AM), esculin (ES), salicin (SA), lactose (Lac), or glucose (Glu), or no carbon source was added to the culture medium. Data were obtained by using RNA-Seq analysis. Annotations are colored according to the cluster of orthologous group classification. The differential normalized transcripts per million (TPM) ratios (DE) are colored according to level: red, DE ≥4; light red, DE ≥2; white, 1.9 ≥ DE ≥ −1.9; light blue, −2 ≥ DE; blue, −4 ≥ DE. Download TABLE S3, XLSX file, 0.1 MB. Copyright © 2017 Theilmann et al. 2017 Theilmann et al. This content is distributed under the terms of the Creative Commons Attribution 4.0 International license.

**TABLE 1:** Highly upregulated genes in the transcriptome of *L. acidophilus* NCFM grown on amygdalin, esculin, or salicin

Locus tag	Annotation^a^	COG^b^	Log_2_ ratio^c^		
			Amy/Glc	Esc/Glc	Sal/Glc
LBA0227	PTS EIIC	G	9.9	0.8	0.9
LBA0725	PTS EIIABC	G	9.7	9.8	8.9
LBA0726	Phospho-β-glucosidase (GH1)	G	7.2	6.9	6.2
LBA1436	Glycerol uptake facilitator protein	G	7.2	4.9	3.8
LBA0631	Hypothetical protein		7.2	2.9	2.9
LBA1435	Hypothetical protein	S	7.1	5	3.6
LBA1434	Dihydroxyacetone kinase	G	6.7	4.5	3.3
LBA1869	β-Phosphoglucomutase	R	6.7	4.2	2.4
LBA1684	PTS EIIA	G	6.6	2.9	2.6
LBA0225	Phospho-β-glucosidase (GH1)	G	6.5	−0.2	−0.1
LBA0724	Transcriptional regulator (antiterminator)	K	6.4	5.5	5.3
LBA0228	Transcriptional regulator	G	6.3	0.9	0.1
LBA1433	Dihydroxyacetone kinase	G	6	3.7	2.7
LBA0728	Hypothetical protein	R	6	4.8	4.1
LBA0555	Myosine-cross-reactive antigen/fatty acid hydratase	S	6	2	1.4
LBA1974	Pyruvate oxidase	E	5.5	3.6	1.8
LBA1689	Isomaltose-6′-phosphate glucosidase (GH4)	G	5.3	1.8	3.7
LBA1812	α-Glucosidase II (GH31)	G	5.3	2.8	2.2
LBA1701	Melibiose operon regulatory protein	K	5.3	4.9	1
LBA0466	Phosphoenolpyruvate carboxykinase (ATP)	C	5.2	2	1.3
LBA0492	Hypothetical protein		5	3.7	2
LBA0606	PTS EIIBC	G	4.9	2.8	2.5
LBA0491	PTS EIIC	G	4.7	3.4	1.5
LBA1797	Hypothetical protein		4.7	2.6	1.3
LBA0877	PTS EIIA	G	4.6	3	1.3
LBA1873	Acetate kinase	C	4.6	0.8	1.2
LBA1709	Mucus binding protein precursor		4.5	3.6	0.5
LBA1632	NAD-dependent aldehyde dehydrogenase	C	4.4	3.3	2
LBA1401	Peroxidase (Npx)	R	4.4	3.1	2.7
LBA0876	PTS EIIC	G	4.4	2.9	2.4
LBA1871	Maltogenic α-amylase (GH13)	G	4.3	2.1	0.9
LBA1411	Fumarate reductase flavoprotein subunit	C	4	1.7	1.4

^a^ Annotations are based on homology or functional characterization when possible.

^b^ COG, cluster of orthologous group classification; C, energy production and conversion; E, amino acid metabolism and transport; G, carbohydrate metabolism and transport; K, transcription; R, general functional prediction only; S, function unknown.

^c^ Differential transcription log_2_ ratio of normalized transcripts per million relative to glucose. Amy, amygdalin; Esc, esculin; Sal, salicin; Glc, glucose. The included genes displayed log_2_ differential expression ratios (of the normalized transcripts per million) of ≥4 for the plant glycosides.

Carbohydrate metabolism and transport genes comprised about one-third of the differential transcriptome. Notably, three genes encoding α-glucan utilization enzymes were highly upregulated, including a GH31 putative α-glucosidase, a GH4 putative isomaltose-6′-phosphate α-glucosidase (LBA1689), which likely confers the breakdown of isomaltose (26), and a putative maltogenic α-amylase (LBA1871), which resides in the maltodextrin utilization cluster (29) (Table 1). The physiological significance of such upregulation is unclear, but α-glucans from starch breakdown by humans and bacteria are common metabolic resources in the small intestine, which is inhabited by lactobacilli (30), and this may explain the observed response. The transcriptional response also revealed the upregulation of genes encoding proteins predicted to be associated with mucus, fibrinogen, and epithelial cell adhesion, e.g., LBA0649, LBA1392, LBA1633, and LBA1709 (Table 1; Table S3) (24, 31, 32). Interestingly, genes encoding cellular defense redox enzymes, e.g., a peroxidase (LBA1401) and an oxidoreductase (LBA1025), were also upregulated, indicating a possible xenobiotic stress response (Table 1; Table S3). Multidrug efflux ABC export systems were also upregulated, e.g., LBA0574 and LBA0575, together with 41 hypothetical proteins (Table S3). Growth on PG appeared to promote increased host interaction and adhesion, which was also observed for *L. rhamnosus* after pretreatment with the PG rutin and phloridzin (33).

### Specific phosphotransferase uptake systems and specialized phospho-β-glucosidases are essential for growth on plant β-glucosides.

**FIG 2:**
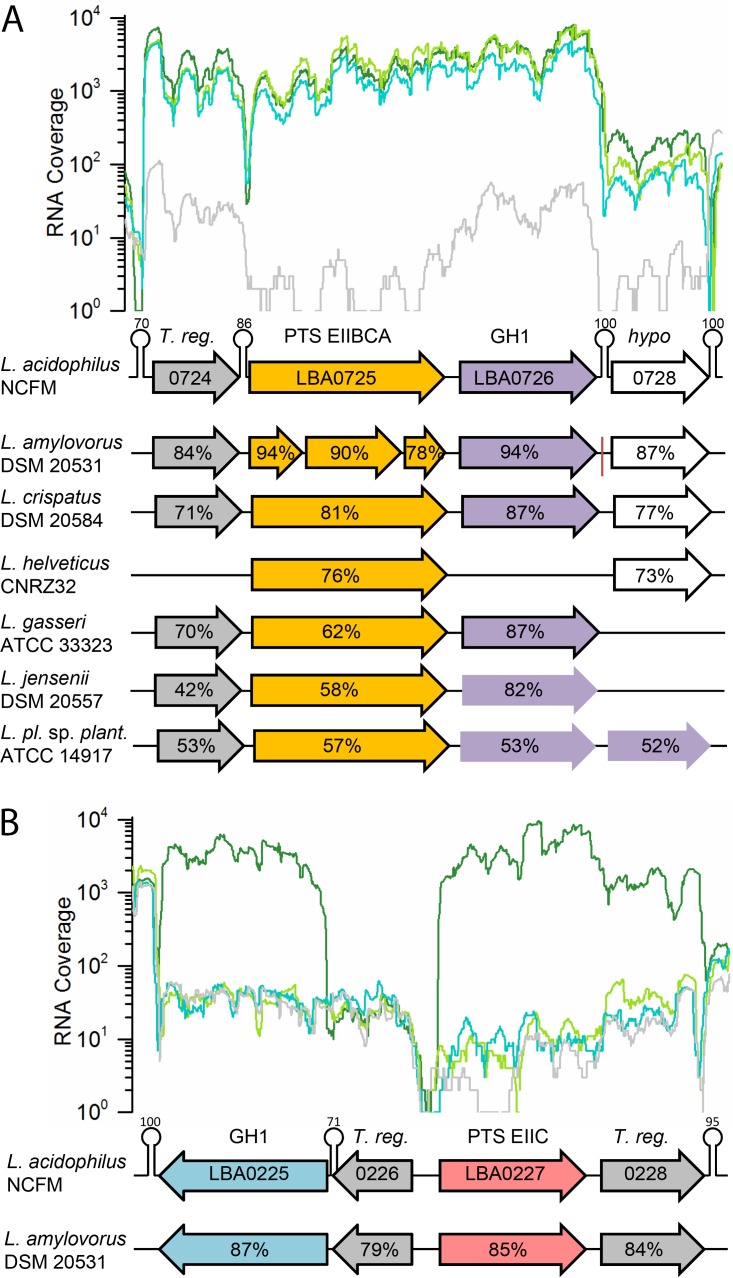
Transcriptional profiles and conservation of plant glycoside utilization loci. The RNA read coverages for amygdalin (dark green), esculin (light green), salicin (turquoise), and glucose (light gray) are shown. (A) The top upregulated locus in *L. acidophilus* NCFM on the three plant glycosides encodes a transcriptional regulator (LBA0724), a PTS EIIBCA transporter (LBA0725), a phospho-β-glucosidase of glycoside hydrolase family 1 (LBA0726), and a hypothetical protein (LBA0728). (B) A locus upregulated exclusively during growth on amygdalin also encodes a P-Bgl (LBA0225), a PTS EIIC transporter (LBA0227), and two transcriptional regulators (gray). Conservation of the loci in selected lactobacilli from the *L*. *delbrueckii* group and the amino acid sequence identities relative to *L. acidophilus* NCFM are shown. The red vertical line signifies the scaffold border. Predicted rho-independent transcriptional terminators are shown as hairpin loops, with overall confidence scores (ranging from 0 to 100) (67).

Growth on PGs highly upregulated two gene loci compared to growth on glucose (Table 1; Table S3), and these findings were also corroborated by quantitative reverse transcriptase PCR (qRT-PCR) analysis (data not shown). The first locus encompassed four genes which were highly upregulated (log_2_ ratios of 4.1 to 8.9, corresponding to 17- to 478-fold upregulation) for all 3 PG. The genes encode a LicT transcriptional antiterminator (LBA0724), an EIIABC component of a phosphotransferase system (PTS; LBA0725), a phospho-β-glucosidase (P-Bgl; LBA0726) of glycoside hydrolase family 1 (GH1) according to the CAZy database (34), and a hypothetical protein (LBA0728) (Fig. 2A). These genes, except for the less frequently transcribed LBA0728, which belongs to the *Lactobacillus* core genome (35), are among the top 10% most upregulated genes in the PG transcriptomes (Table S3). The second locus, which was only transcriptionally responsive to amygdalin, encodes another P-Bgl of GH1 (LBA0225), a divergently transcribed PTS enzyme II component (EIIC; LBA0227), and a transcriptional regulator (LBA0228) (Fig. 2B). Both these gene loci are strictly conserved in the *L. acidophilus* species and to some extent in related lactobacilli from the *Lactobacillus delbrueckii* group (Fig. 2; Table S4).

10.1128/mBio.01421-17.6 TABLE S4  Conservation of plant glycoside utilization gene loci identified in this work (annotated by their locus tags and accession numbers) in *Lactobacillus acidophilus* strains in the NCBI organism database. The table shows the amino acid identities and also the sequence coverage if it was less than 100% of the protein in *L. acidophilus* NCFM. Download TABLE S4, DOCX file, 0.02 MB. Copyright © 2017 Theilmann et al. 2017 Theilmann et al. This content is distributed under the terms of the Creative Commons Attribution 4.0 International license.

**FIG 3:**
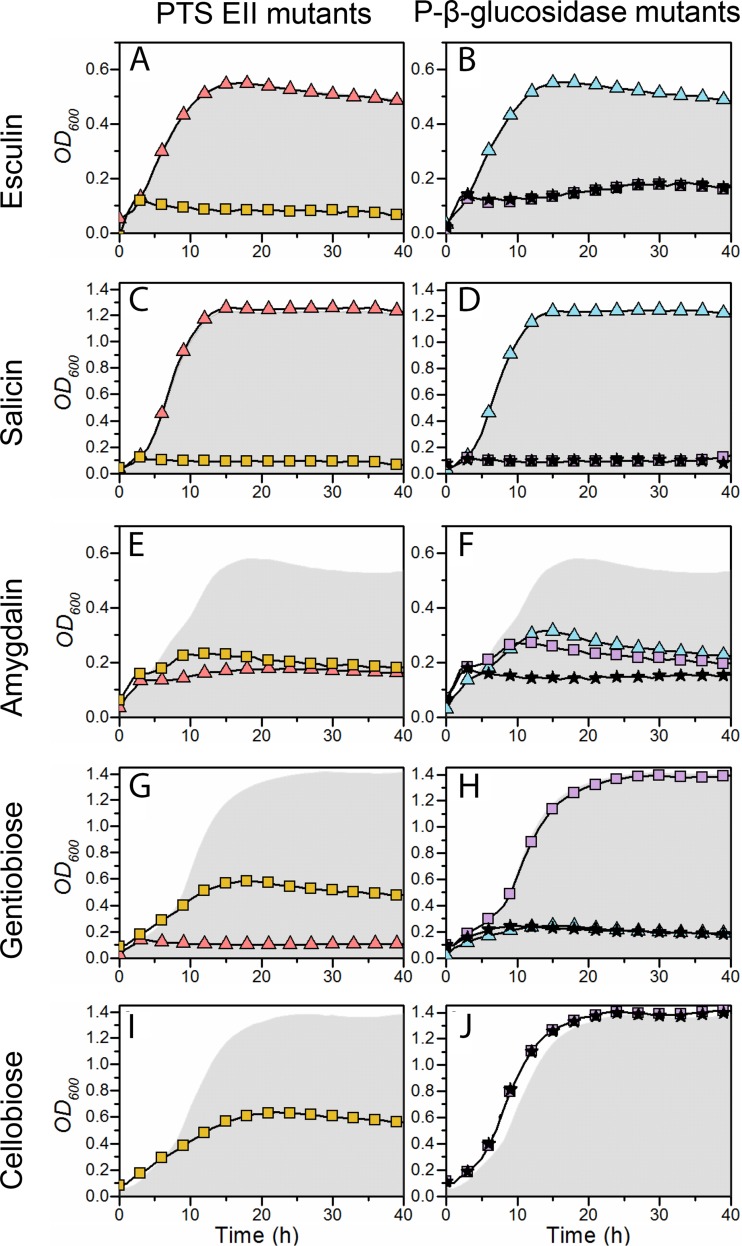
Phenotypic growth analyses of deletion mutants of EII PTS transporters (left) and phospho-β-glucosidases (right) on the β-glucosides esculin, salicin, and amygdalin, and the disaccharides gentiobiose and cellobiose. The background *Δupp* strain is shown in gray, and the growth of the mutant strains is shown for PTS EIIC (LBA0227; pink triangle), the phospho-β-glucosidase (LBA0225; light blue triangles), the PTS EIIABC (LBA0725; yellow squares), the second phospho-β-glucosidase (LBA0726; lilac squares), and the double phospho-β-glucosidase mutant (LBA0225/LBA0726; black stars). The color scheme is consistent with that used for the gene loci in Fig. 2. The growth experiments were performed in biological triplicates, and the average errors were <6%.

To establish the functional significance of these two loci, constructs with single deletions of each PTS EII and P-Bgl gene or a double deletion of both P-Bgl genes were created by using the *upp-*based counterselectable gene replacement system (36) (Table S5), and the growth phenotypes of the mutant strains were analyzed (Fig. 3). This analysis is very powerful, particularly as the physiological 6′-phosphorylated substrates of the GH1 enzymes are not available to perform enzymatic analyses *in vitro*. The growth of the *ΔLBA0725* mutant (with an inactive PTS EIIABC) was abolished on esculin or salicin, severely reduced on amygdalin, and moderately reduced on cellobiose or gentiobiose. The abolished growth on esculin and salicin identified this EIIABC as the sole transporter for these PG, but the reduced growth on the other compounds suggested additional roles for this transport system. The growth profile of the *ΔLBA0726* mutant, which lacks a functional P-Bgl, was similar on PGs, but growth on either cellobiose or gentiobiose was unaffected. This phenotype also supports the specificity of P-Bgl (LBA0726) toward the PGs esculin and salicin (Fig. 3). Accordingly, we can assign the specificity of this locus to the uptake and hydrolysis of PGs, with a preference for distinct monoglucosylated small aromatic aglycones.

10.1128/mBio.01421-17.7 TABLE S5  Strains used and constructed for gene deletion mutants in *L. acidophilus* NCFM. Download TABLE S5, DOCX file, 0.02 MB. Copyright © 2017 Theilmann et al. 2017 Theilmann et al. This content is distributed under the terms of the Creative Commons Attribution 4.0 International license.

The growth of the *ΔLBA0227* mutant (inactive EIIC) in the second locus, which was exclusively upregulated by amygdalin, was abolished on both amygdalin and gentiobiose (Fig. 3E and G), both of which share a β-(1,6)-diglucoside moiety (Fig. 1). The phenotypes for salicin and esculin were invariant compared to the wild-type strain (Fig. 3A and C). These data provided compelling evidence for the specificity of this PTS EIIC transporter for amygdalin and gentiobiose, consistent with previously reported upregulation in response to gentiobiose (26). This specificity is also supported by the phenotype of the P-Bgl mutant *ΔLBA0225*. The severe reduction in growth for the *ΔLBA0726* mutant lacking the P-Bgl from the first locus on amygdalin (Fig. 3F), but not on gentiobiose (Fig. 3H), suggests a role for this enzyme in the catabolism of amygdalin. Indeed, growth on amygdalin was only abolished with the double P-Bgl mutant (Fig. 3F). The identification of low levels of prunasin, the singly deglucosylated form of amygdalin (Table S6), suggests that the deglycosylation of amygdalin occurs in two steps, with sequential cleavage of the nonreducing β-(1,6)-linked glucosyl by the P-Bgl that recognizes the β-(1,6)-gentiobiose moiety (LBA0225) and by a second P-Bgl that cleaves monoglucosylated compounds (LBA0726) to release the aglycone moiety. Based on these data, we can assign the specificity of the locus encoding the PTS EIIC transporter (LBA0227) and the phospho-β-glucosidase (LBA0225) to compounds with a β-(1,6)-diglucoside motif, like gentiobiose and amygdalin. The full deglycosylation of PGs possessing a gentiobiose moiety like amygdalin, however, required the additional activity of the second P-Bgl (LBA0726).

10.1128/mBio.01421-17.8 TABLE S6  Plant glycosides and their metabolites in *L. acidophilus* NCFM culture supernatants. Supernatants were analyzed by UHPLC-qTOF-MS. The starting plant glycoside substrates, which were identified in the cultures before inoculation, are shown in bold and underlined. The aglycones are shown in bold. The metabolite analyses were carried out from the 24-h culture supernatant samples. Download TABLE S6, DOCX file, 0.02 MB. Copyright © 2017 Theilmann et al. 2017 Theilmann et al. This content is distributed under the terms of the Creative Commons Attribution 4.0 International license.

### *L. acidophilus* cells externalize the bioactive aglycones of PGs and preferentially utilize glucosides that support the highest growth levels.

We monitored the growth of *L. acidophilus* NCFM and analyzed the metabolites in the culture supernatants at 0 and 24 h. The PG were identified in the preculture medium (Table S6). Depletion of the PGs that supported growth (Fig. 1) was proportional to growth (the final OD_600_), and the respective aglycones lacking the glucosyl moiety (loss of 162 Da) (Table S6) were identified in the culture supernatants. The growth on polydatin was verified from the extent of depletion (Fig. 1), the identification of the aglycone resveratrol (Table S6), and the production of lactate. The only deviation from this trend was the absence of the aglycone of amygdalin (mandelonitrile). Instead, the main metabolite of amygdalin utilization was benzaldehyde, which was only detectable by UV, due to its volatility. The PGs that did not support growth persisted, and no metabolites of these PGs were detected at 24 h.

**FIG 4:**
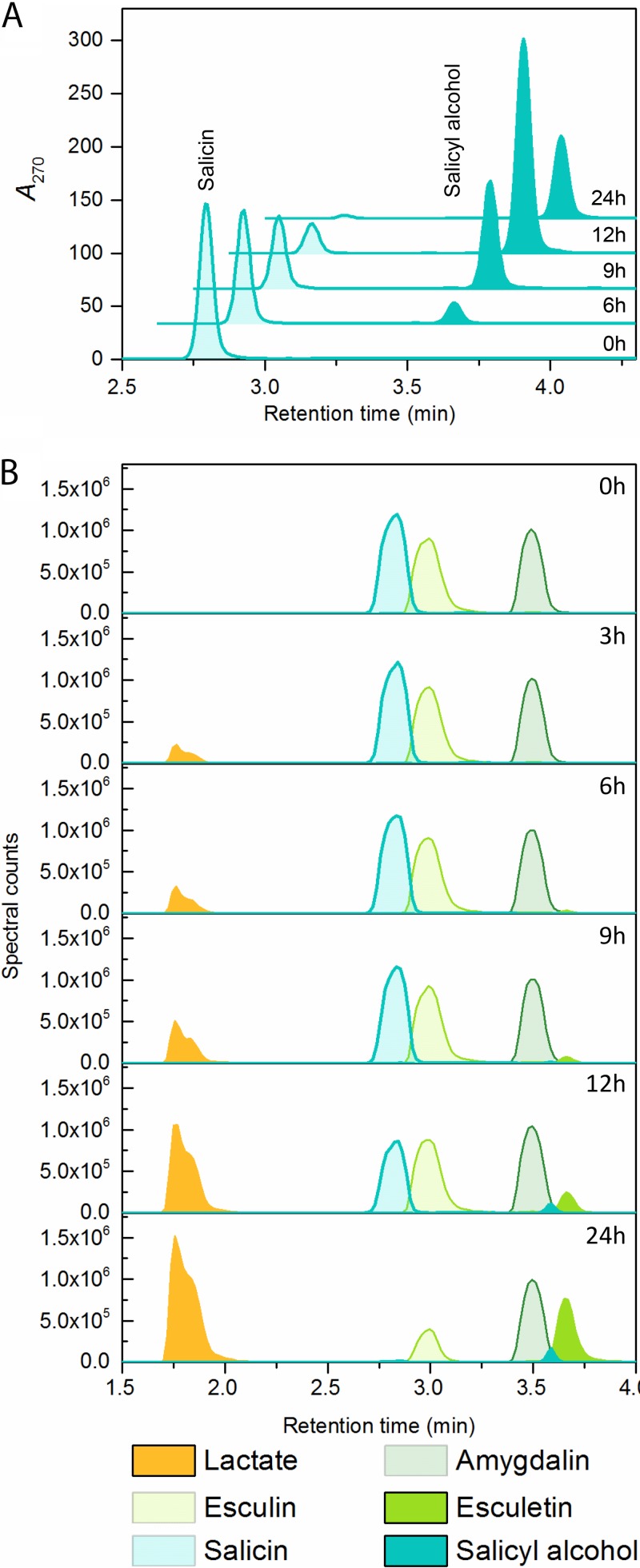
Time-resolved metabolite analysis of *L. acidophilus* NCFM grown on plant glucosides. (A) Time course depletion of salicin and appearance of its aglycone salicyl alcohol in the culture supernatants, visualized as the area under the *A*_270_ peaks in the UHPLC–qTOF-MS chromatograms. (B) Preference of *L. acidophilus* NCFM for plant glycosides during growth on an equimolar mixture of salicin, esculin, and amygdalin. Salicin was preferred, followed by esculin, while amygdalin was hardly consumed after 24 h. The aglycones of the plant glycosides and the concentration of lactate increased concomitantly with growth.

We also monitored the temporal changes in concentrations of the three most available PGs, salicin, esculin, and amygdalin, and of their metabolites in culture supernatants. The concentration of salicin decreased throughout the growth period (Fig. 4A; Fig. S1), while an inverse trend was observed for the aglycone salicyl alcohol during exponential-phase growth. Notably, the aglycone moiety of salicin *per se* was unable to support growth of *L. acidophilus* (data not shown). The same pattern was observed for esculin, which was depleted concomitantly with the increase in the concentration of the aglycone metabolite esculetin during the first 12 h of growth (Fig. S1).

10.1128/mBio.01421-17.1 FIG S1  Results of the time-resolved metabolite analysis of salicin (left), esculin (middle), and amygdalin (right) in *L. acidophilus* NCFM culture supernatants, analyzed by UHPLC-qTOF-MS. The growth (measured as the OD_600_ and shown as light gray background) together with the concentrations of plant glycosides (full circles) and their main metabolites (hollow circles) are shown. Download FIG S1, PDF file, 0.3 MB. Copyright © 2017 Theilmann et al. 2017 Theilmann et al. This content is distributed under the terms of the Creative Commons Attribution 4.0 International license.

The concentration of amygdalin in the culture supernatant also decreased steadily, concomitant with an increase in benzaldehyde (Fig. S1). In contrast to the other 2 PGs, however, only about one-third of the initial amygdalin was utilized during 24 h of growth, and the summed concentration of amygdalin and benzaldehyde was invariant over time. Low levels of the monodeglucosylated metabolite of amygdalin, prunasin (37), were identified (Table S6). Although the corresponding aglycone, mandelonitrile, was identified in the first 6 h ([M + CH_3_COO]^−^ adduct; *m/z* 192.0664), the main amygdalin metabolite was benzaldehyde, which is produced via a hydrogen cyanide elimination reaction of mandelonitrile. This reaction is catalyzed by nitrile lyase but has also been reported to occur spontaneously (38). This is the likely scenario for our experiment, as no nitrile lyase is encoded by *L. acidophilus*. Detection of traces of scopoletin, the methylated form of the esculin aglycone (Table S6), was the only evidence for enzymatic modification of the aglycones of PG, but the paucity of this species casts doubt on the specificity of this modification. Taken together, our metabolite analyses are supportive of *L. acidophilus* largely exporting noncarbohydrate moieties without enzymatic modification. The mechanism of externalization is not clear, but the export systems, e.g., an ATP-binding cassette exporter in the case of esculin (LBA0573 to LBA0575), are upregulated in the transcriptome (Table S3).

To evaluate whether amygdalin, esculin, and salicin are taken up randomly or according to a certain preference, we analyzed the supernatants of *L. acidophilus* NCFM grown on equimolar concentrations of these PG. Strikingly, salicin was the first compound to be fully depleted, followed by esculin, whereas significant amounts of amygdalin persisted after 24 h of growth (Fig. 4B), thus establishing the clear preference of *L. acidophilus* in the utilization of PG that support its best growth.

## DISCUSSION

A considerable proportion of the thousands of diet-derived known phytochemicals mediates positive health effects in humans (39). The daily intake of phytochemicals is relatively high due to the enrichment of common nutritional sources, such as fruits, berries, nuts, vegetables, herbs, and beverages such as wine and tea (13). Frequently, phytochemicals occur as glycoconjugates that exhibit lower bioactivity and bioavailability than their aglycone derivatives, which are smaller in size and typically less polar (40, 41). Therefore, deglycosylation of PGs is likely an important factor in modulating their biological activity (42). The health impact of HGM-mediated biotransformation of drug xenobiotics and diet-derived phytochemicals has gained considerable interest (4, 43). Our insight into this latter metabolic aspect of the HGM is conspicuously limited. The small intestine is the primary site for absorption of nutrients and xenobiotics, which lends extra gravity to the metabolic activities of HGM prevalent in this part of the gastrointestinal tract, where lactobacilli constitute an important part of the microbial population (11). Based on our findings, we report here the versatility of the probiotic bacterium *L. acidophilus* NCFM in utilization of dietary therapeutically active PG, revealing that only the carbohydrate moieties are catabolized while the aglycones are externalized, making them bio-accessible to absorption by the host or available for further interactions with other organisms of the HGM.

**FIG 5:**
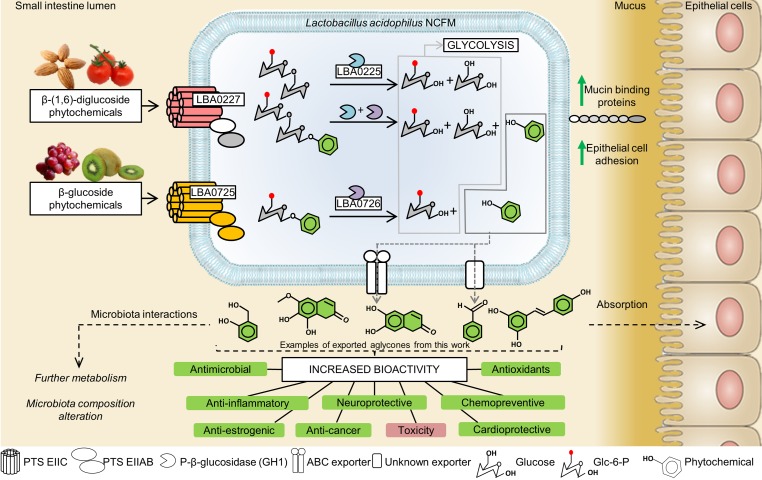
Plant glucoside utilization model for *L. acidophilus* and closely related human gut lactobacilli. Plant glucosides consisting of mono- or bicyclic aromatic rings conjugated with a glycosyl moiety and present in the diet are taken up by dedicated PTS transporters and hydrolyzed by specific phospho-β-glucosidases into the glycolytic precursors glucose-6-phosphate and glucose. The aglycones are exported likely by an upregulated ATP-binding cassette exporter or by other unknown transporters, rendering them accessible for absorption by the human host and eliciting various biological activities, most of which are beneficial. Antimicrobial effects and subsequent changes in the microbiota composition are common effects of the aglycones, but further modifications and modulations of biological activity have been reported (4, 43, 49). The solid arrows show the steps established in our study, whereas the dashed lines indicate physiological effects that have been reported in the literature.

Carbohydrates are mainly taken up by PTS transporters in lactobacilli (28, 44). Translocation is coupled to phosphorylation of the glycoside mostly at the 6′-position via an enzymatic cascade that relays the phosphoryl group to a substrate-specific EIIC complex (45). The EIIC forms the translocation channel that defines the specificity of the EII complex. Phosphorylation is relayed via EIIA and EIIB enzymes, of which the latter is known to interact specifically with EIIC. The EII modules are either encoded by a single gene, e.g., the gene for the EIIABC salicin and esculin uptake system (LBA0725), or by 2 to 3 separate genes, to assemble the phosphorylation cascade. The amygdalin EIIC component (LBA0227) requires coupling from EIIA and EIIB modules that are not encoded by the same locus. This EIIC is only upregulated upon growth on its substrate, amygdalin, whereas the LBA0725 EIIABC is highly upregulated during growth on the substrates salicin and esculin, as well as on amygdalin (Table 1; Fig. 3 and 5). Accordingly, inactivation of the EIIC elicits an impaired growth phenotype only on the substrate amygdalin, whereas the inactivation of the EIIABC causes an approximate 50% reduction of growth on amygdalin, as well as on the two disaccharides cellobiose and gentiobiose, both of which are not hydrolyzed by the P-Bgl encoded by this locus (Fig. 3). The lack of growth on amygdalin or gentiobiose, when the EIIC system is inactivated, precludes uptake of these compounds solely via the EIIABC system. A possible rationale for the coregulation of the two transporters and the phenotypic impact of the EIIABC on nonsubstrates is that the EIIA and/or EIIB components of LBA0725 contribute in coupling phosphorylation to the amygdalin EIIC system and possibly to other EIIC modules. The less drastic phenotype of EIIABC on nonsubstrates, however, suggests that the contribution of this transporter can be complemented by other PTS systems. To our knowledge, this functional overlap between PTS systems that are assigned to different families (46) has not been reported before and merits further investigation. Such an overlap may orchestrate interplay between different transporters to confer the uptake of diverse sugars by bacteria.

There is a large and growing body of evidence on functionalities of phytochemicals and their beneficial health effects (i.e., nutraceuticals) (39, 47, 48). Several intervention studies have shown changes in HGM composition, especially an increase in lactobacilli and bifidobacteria, due to phytochemicals, which are also suggested to have antimicrobial effects (7, 49, 50). The routes of conversion of these compounds, however, have not been addressed at the molecular level. Our data suggest an important role of human gut *L. acidophilus* in the activation of dietary-relevant PG (Fig. 5). For example, salicin, the best growth substrate for *L. acidophilus* NCFM in this study, is a pharmacologically inactive precursor of the analgesic and antirheumatic drug salicylic acid. Indeed, salicylic acid has been reported to be the main metabolite (86%) in serum after oral administration of salicin-rich willow bark extract in humans (51). Our study revealed that *L. acidophilus* performs a step in this bioactivation, via deglycosylation and externalization of salicyl alcohol, which becomes accessible for oxidation to salicylic acid performed by other microbiota. Fraxin, which also sustains the growth of *L. acidophilus*, is one of the active ingredients in some Chinese and Japanese herbal medicines and has several potential positive health effects, including protection against oxidative stress (52). *L. acidophilus* also converts polydatin, which is enriched in wines and tea, to resveratrol, which is one of the most-studied therapeutic phytochemicals due to its implication in protection against, e.g., inflammation, cancer, and obesity (53–55). Other lactobacilli have also been implicated in the metabolism of other PGs, e.g., the *in vitro* conversion of the isoflavonic daidzin, present in soy products, by the pig intestinal commensal *Lactobacillus mucosae* EPI2, to the estrogen-mimicking aglycone equol, which has been proposed to be protective against breast cancer (56, 57).

*In silico* analysis of genomic sequences of *L. acidophilus* strains revealed the conservation of the PG utilization loci identified in the present study, indicating the potential ability of this species to metabolize PGs (Table S4). Our growth survey using four different PG revealed large species variations in growth (Table S2). Generally, *L. acidophilus* strains were among the top strains in terms of growth on PG, and lactobacilli strains from the gut appeared to better at PG utilization than counterparts from other ecologic niches, suggesting a competitive advantage in the adaptation to the human gut environment. Gene landscape analyses showed a correlation between growth on salicin or esculin and the presence of the intact LBA0724-LBA0726 locus in the tested strains that belong to the taxonomically closely related *L. delbrueckii* clade, i.e., *L. acidophilus*, *Lactobacillus crispatus*, *Lactobacillus jensenii*, and *Lactobacillus gasseri* (58) (Fig. 2A). Strains missing one or more genes within this cluster or which have a fragmented version of the LBA0725 transporter gene were conversely unable to grow on esculin or salicin (Table S2). Growth on amygdalin is less common within the *L. delbrueckii* group, in line with the limited occurrence of the amygdalin gene cluster (LBA0225 to LBA0228). The *L. amylovorus* strain has a fragmented and likely dysfunctional version of EIIABC LBA0725 (Fig. 2A, total sequence coverage of 72%). This strain is unable to grow on amygdalin, consistent with the involvement of modules from this PTS in the uptake of β-(1,6)-glucosides, as discussed above. The good growth of more distant species, e.g., *Lactobacillus plantarum* subsp. *plantarum* and *Lactobacillus rhamnosus* GG, which lack the gene locus, discloses the presence of alternative routes for the utilization of amygdalin in other *Lactobacillus* clades. The functional data in the present study, combined with the genetic analysis, provide an extended predictive power for PG utilization within closely related lactobacilli, although the metabolic pathways and transporters are likely to be diverse across a larger taxonomic group. Although our limited screening was not sufficient to establish PG utilization in other microbiota taxa, our results do hint at the specialization of *L. acidophilus* growth on PGs that are not utilized by other common taxa (Fig. S2).

10.1128/mBio.01421-17.2 FIG S2  Growth on plant glycosides of common human gut microbiota commensals from the *Bifidobacterium* (Bi), *Bacteroides* (Ba), and *Roseburia* (R) genera on plant glycosides. The strains were grown in modified MRS medium, except for *Roseburia intestinalis*, which was grown in YCFA medium (1). The media were supplemented with 0.5% (wt/vol) of the respective substrate, and a control without any supplementation was included (no carb). Growth (measured based on the OD_600_) was determined after 24 h of growth. The reported growth data are means (with standard deviations) of biological duplicates. Download FIG S2, PDF file, 0.1 MB. Copyright © 2017 Theilmann et al. 2017 Theilmann et al. This content is distributed under the terms of the Creative Commons Attribution 4.0 International license.

Taken together, this study enabled the elucidation of a novel pathway for the bioconversion of PGs and the externalization of their bioactive aglycones by the human gut-adapted *L. acidophilus* and closely related taxa. The bioconversion of PG is accompanied by a modulation of the activities of the phytochemicals in the small intestine, which renders these compounds bioavailable for further functional interplay with the host and other HGM taxa (Fig. 5). In conclusion, this study casts light on underexplored facets of the metabolism of plant-derived glycosides and their bioconversion by the microbiota that exert a significant impact on human health. Further work is required to bring insight on the fate of PG in the human gut ecologic niche and to evaluate the clinical and possible therapeutic implications of PG bioconversion by the HGM.

## MATERIALS AND METHODS

### Chemicals and carbohydrates.

The plant glycosides utilized in this work are described in Table S1. All other chemicals used were of high purity.

### Bacterial strains and growth.

Bacterial strains and plasmids used in this study are presented in Table S5. *Lactobacillus* strains were propagated statically in de man-Rogosa-Sharpe (MRS) broth (Difco Laboratories, Detroit, MI) under aerobic conditions or on MRS agar plates (1.5% [wt/vol]; Difco) under anaerobic conditions at 37°C, or at 42°C for pTRK669 elimination (59). Recombinant *L. acidophilus* strains were selected in the presence of 2 μg ml^−1^ erythromycin (Sigma-Aldrich, St. Louis, MO, USA) and/or 2 to 5 μg ml^−1^ chloramphenicol (Sigma). Selection of plasmid-free double recombinants was done on a semidefined agar medium containing 2% (wt/vol) glucose (GSDM) (60) with 100 μg ml^−1^ 5-fluorouracil (5-FU; Sigma), as described by Goh et al. (36).

For initial growth and gene expression studies, *L. acidophilus* NCFM was propagated three times in semidefined medium supplemented with either 1% or 0.5% (wt/vol) of the plant glycoside or carbohydrate (Table S1). The plant glycoside screening was carried out in at least biological triplicates. For the RNA-Seq analysis, cells were harvested by centrifugation (3,220 × *g*, 10 min, 25°C) in the mid-exponential phase (OD_600_, 0.6 to 0.8) and stored at −80°C for subsequent RNA isolation. For the mass spectrometry metabolite analyses, 200-μl samples were taken at 0, 3, 6, 9, 12, and 24 h of growth, cells were removed by centrifugation, and supernatants were stored at −80°C for further analysis.

Phenotypic growth assays were performed using 1% (vol/vol) overnight cultures of *L. acidophilus* strains (Table S5) and other *Lactobacillus* species (Table S2) grown on SDM supplemented with 1% (wt/vol) glucose to inoculate 200 μl of SDM supplemented with 1% (wt/vol) of the examined carbohydrate (0.5% in the case of esculin) in 96-well microplate wells (Corning Costar, Corning, NY). Phenotypic growth assays of *L. acidophilus* NCFM and its knockout variants were performed in biological triplicates, whereas the growth screening of other *L. acidophilus* strains or other *Lactobacillus* species was performed in biological duplicates. The microplates were sealed with clear adhesive film and incubated at 37°C in a Fluostar Optima microplate reader (BMG Labtech, Cary, NC), and the culture OD_600_ was monitored for 30 h.

*Escherichia coli* EC101, used for generating the *L. acidophilus* gene knockouts, was grown in brain heart infusion (BHI) broth (Difco) at 37°C with aeration in the presence of kanamycin (40 μg ml^−1^). Recombinant *E. coli* EC101 cells containing pTRK935-based plasmids were selected with erythromycin (150 μg ml^−1^). Growth of *Bifidobacterium longum* subsp. *longum* DSM 20219, *Bifidobacterium longum* subsp. *infantis* DSM 20088, and *Bacteroides ovatus* DSM 1896 was carried out in MRS medium or modified MRS medium supplemented with a 1% (wt/vol) carbon source. *Roseburia intestinalis* L1-82 was cultured in yeast extract-casein hydrolysate-fatty acids (YCFA) medium supplement with a carbon source (61).

### RNA extraction, sequencing, and transcriptional analysis.

Pellets from 10-ml cell cultures were resuspended in 1 ml of TRI reagent (Thermo Fisher Scientific, Waltham, MA) and thereafter transferred into 1.5-ml bead beating conical tubes with 0.1-mm glass beads (BioSpec Products, Inc., Bartlesville, OK), and cells were disrupted by six 1-min cycles (with 1 min on ice intermittently) with a Mini-Beadbeater 16 apparatus (BioSpec Products). RNA purification was performed using the Direct-zol RNA MiniPrep kit (Zymo Research, Irvine, CA) with on-column DNase I treatment followed by an additional Turbo DNAse (Thermo Fisher) treatment of the eluted RNA, and further purification was carried out using the RNA Clean and Concentrator 5 kit (Zymo Research). The quality of RNA was analyzed using an Agilent 2100 Bioanalyzer (Agilent Technologies, Santa Clara, CA), and the absence of genomic DNA was confirmed by PCR using *L. acidophilus* NCFM gene-specific primers. Library preparation and RNA sequencing were performed by the High-Throughput Sequencing and Genotyping Unit of the Roy J. Carver Biotechnology Centre, University of Illinois (Urbana-Champaign, IL). After rRNA removal (using a Ribo-Zero rRNA removal kit for bacteria; Illumina, San Diego, CA), library preparation was carried out using the TruSeq stranded total RNA library prep kit (Illumina). Single-read RNA sequencing was performed using a HiSeq 2500 ultrahigh-throughput sequencing system (Illumina) and the Illumina HiSeq SBS v4 kit (Illumina) with a read length of 160 nucleotides (nt). The raw reads were demultiplexed with the bcl2fastq conversion software (v 2.17.1.14; Illumina), trimmed for the adapter sequences, quality trimmed to remove sequence reads with an error probability threshold of 0.001 (Phred score, 30), and filtered to remove reads of <20 nt by using Geneious version 9.0.4 (62). The quality of the reads was assessed by using FastQC v0.11.5 (http://www.bioinformatics.babraham.ac.uk/projects/fastqc/). The resulting reads were then mapped to the *L. acidophilus* NCFM reference genome by using the Geneious Mapper with default settings (62). The sequencing coverage depths were calculated to be 610× to 692×, and transcriptional analyses were based on the number of normalized transcripts per million (nTPM), calculated within Geneious. Differentially expressed genes were defined as having a log_2_ ratio of ≥2 unless otherwise stated.

### RT-qPCR assay.

To confirm the results of the RNA-Seq transcriptional study, RT-qPCR analysis of selected genes was performed (63). Briefly, the iTaq universal SYBR green one-step kit (Bio-Rad Laboratories, Hercules, CA) was used according to the manufacturer’s instructions, except for scaling down to 25-μl reaction mixtures with 50 ng of RNA template and 300 nM of each primer (Table S7). An iCycler MyiQ single-color detection system (Bio-Rad) was used, and the data were analyzed using iCycler MyiQ software v1.0 (Bio-Rad). The correlation coefficients for the standard curves and PCR efficiencies were between 0.930 and 0.999 and 88.7% to 102.5%, respectively.

10.1128/mBio.01421-17.9 TABLE S7  Primers used in this study. Download TABLE S7, DOCX file, 0.02 MB. Copyright © 2017 Theilmann et al. 2017 Theilmann et al. This content is distributed under the terms of the Creative Commons Attribution 4.0 International license.

### DNA manipulation and transformation.

Genomic DNA from *L. acidophilus* NCFM and mutants thereof was isolated using the ZR fungal/bacterial DNA MiniPrep kit (Zymo Research). Plasmid DNA was isolated using the QIAprep Spin MiniPrep kit (Qiagen, Hilden, Germany). Restriction enzymes were from Roche (Basel, Switzerland), and T4 DNA ligase was from NEB (New England Biolabs, Ipswich, MA). *PfuUltra II* fusion HS DNA polymerase (Agilent Technologies, Santa Clara, CA) was used for cloning, and Choice-*Taq* Blue DNA polymerase (Denville Scientific, South Plainfield, NJ) was used for PCR screening of recombinants. PCR amplicons were analyzed on 0.8% (wt/vol) agarose gels and extracted using the QIAquick gel extraction kit (Qiagen). DNA sequencing was performed by Eton Biosciences (Durham, NC).

### Construction of gene deletion mutants.

The *L. acidophilus* NCFM genes LBA0225 and LBA0726, both of which encode P-Bgl of glycoside hydrolase family 1 (GH1) enzymes (34), in addition to the LBA0227 and LBA0725 genes which encode EIIC and EIIABC components of two PTS, respectively (64), were deleted using the *upp-*based counterselectable gene replacement system (36). Briefly, in-frame deletions were constructed by amplifying 650 to 750 bp of the up- and downstream flanking regions of the deletion targets with two primer pairs, e.g., LBA0225A/LBA0225B and LBA0225C/LBA0225D (Table S7). The resulting purified products were joined by splicing using overlap extension PCR (SOE-PCR) (65) and amplified to establish the deletion alleles. The SOE-PCR products, which included flanking restriction enzyme sites, were cloned within the BamHI and SacI/EcoRI sites of the pTRK935 integration vector and transformed into *E. coli* EC101. The resulting recombinant plasmids (pTRK1113 to -6) were confirmed by DNA sequencing and electroporated into *L. acidophilus* NCK1910 (Table S5), which contains the pTRK669 helper plasmid, and recovery of single- and double-crossover recombinants was performed as previously described (63). Recombinants carrying the new gene deletion alleles were isolated by colony PCR using primer pairs denoted up-down (e.g., LBA0225*up*/LBA0225*down*), which anneal to the flanking regions of the amplicons. Sequence integrity and in-frame deletions were verified by DNA sequencing employing the aforementioned primer pairs and a primer denoted *mid* (e.g., LBA0225*mid*). The mutations were in-frame deletions of 90 to 96% of the coding regions.

### Analysis of plant glycoside uptake from *L. acidophilus* NCFM culture supernatants using mass spectrometry.

The supernatants of *L. acidophilus* NCFM cultures grown on amygdalin, arbutin, esculin, or salicin as carbon sources were analyzed during 24 h by using ultrahigh-performance liquid chromatography–diode array detection–quadruple time of flight mass spectrometry (UHPLC–DAD–Q-TOF-MS). Samples were diluted 1:20 (vol/vol) with methanol, and an injection volume of 1.5 μl was used. Separation was carried out on an Agilent Poroshell 120 phenyl-hexyl column (2.1 by 150 mm, 2.7 μm) using the Infinity 1290 UHPLC system (Agilent Technologies, Santa Clara, CA) equipped with a UV-visible spectrum diode array detector. Separation was performed at 0.35 ml min^−1^, 60°C, with a linear gradient consisting of water (A) and acetonitrile (B), both buffered with 20 mM formic acid, starting at 10% B and increased to 100% in 15 min, at which conditions were held for 2 min, returned to 10% in 0.1 min, and kept for 3 min. MS detection was performed on an Agilent 6550 iFunnel QTOF MS equipped with the Agilent Dual jet stream electrospray ion source with a drying gas temperature of 160°C and gas flow of 13 liters min^−1^, whereas the sheath gas temperature was 300°C and flow was 16 liters min^−1^. Ionization was conducted in ESI− mode with a capillary voltage set to 4,000 V and nozzle voltage set to 500 V. Mass spectra were recorded as centroid data for *m/z* 85 to 1700 in MS mode with an acquisition rate of 10 spectra s^−1^. To avoid carryover, the needle seat was back-flushed for 15 s at 4 ml min^−1^ with each of the following: (i) isopropanol–0.2% ammonium hydroxide (wt/vol) in water (1:1 [vol/vol]); (ii) acetonitrile with 2% formic acid (wt/vol); (iii) water with 2% formic acid. Data were processed with the Agilent MassHunter qualitative analysis B.07.00 software package (Agilent Technologies), and molar concentrations were obtained from standard curves of the plant glycosides and their main metabolites. Targeted compound searches were performed using lists of previously identified compounds plus standard chemical modifications (37, 51, 66).

## References

[1] Charbonneau MR, Blanton LV, DiGiulio DB, Relman DA, Lebrilla CB, Mills DA, Gordon JI 2016 A microbial perspective of human developmental biology. Nature 535:48–55. doi:10.1038/nature18845. 27383979 PMC5358965

[2] Sonnenburg JL, Bäckhed F 2016 Diet-microbiota interactions as moderators of human metabolism. Nature 535:56–64. doi:10.1038/nature18846. 27383980 PMC5991619

[3] Zitvogel L, Ayyoub M, Routy B, Kroemer G 2016 Microbiome and anticancer immunosurveillance. Cell 165:276–287. doi:10.1016/j.cell.2016.03.001. 27058662

[4] Spanogiannopoulos P, Bess EN, Carmody RN, Turnbaugh PJ 2016 The microbial pharmacists within us: a metagenomic view of xenobiotic metabolism. Nat Rev Microbiol 14:273–287. doi:10.1038/nrmicro.2016.17. 26972811 PMC5243131

[5] Martens EC, Kelly AG, Tauzin AS, Brumer H 2014 The devil lies in the details: how variations in polysaccharide fine-structure impact the physiology and evolution of gut microbes. J Mol Biol 426:3851–3865. doi:10.1016/j.jmb.2014.06.022. 25026064 PMC4252772

[6] Scott KP, Gratz SW, Sheridan PO, Flint HJ, Duncan SH 2013 The influence of diet on the gut microbiota. Pharmacol Res 69:52–60. doi:10.1016/j.phrs.2012.10.020. 23147033

[7] David LA, Maurice CF, Carmody RN, Gootenberg DB, Button JE, Wolfe BE, Ling AV, Devlin AS, Varma Y, Fischbach MA, Biddinger SB, Dutton RJ, Turnbaugh PJ 2014 Diet rapidly and reproducibly alters the human gut microbiome. Nature 505:559–563. doi:10.1038/nature12820. 24336217 PMC3957428

[8] Desai MS, Seekatz AM, Koropatkin NM, Kamada N, Hickey CA, Wolter M, Pudlo NA, Kitamoto S, Terrapon N, Muller A, Young VB, Henrissat B, Wilmes P, Stappenbeck TS, Núñez G, Martens EC 2016 A dietary fiber-deprived gut microbiota degrades the colonic mucus barrier and enhances pathogen susceptibility. Cell 167:1339–1353.e21. doi:10.1016/j.cell.2016.10.043. 27863247 PMC5131798

[9] Rogowski A, Briggs JA, Mortimer JC, Tryfona T, Terrapon N, Lowe EC, Baslé A, Morland C, Day AM, Zheng H, Rogers TE, Thompson P, Hawkins AR, Yadav MP, Henrissat B, Martens EC, Dupree P, Gilbert HJ, Bolam DN 2015 Glycan complexity dictates microbial resource allocation in the large intestine. Nat Commun 6:7481. doi:10.1038/ncomms8481. 26112186 PMC4491172

[10] Cockburn DW, Koropatkin NM 2016 Polysaccharide degradation by the intestinal microbiota and its influence on human health and disease. J Mol Biol 428:3230–3252. doi:10.1016/j.jmb.2016.06.021. 27393306

[11] Donaldson GP, Lee SM, Mazmanian SK 2016 Gut biogeography of the bacterial microbiota. Nat Rev Microbiol 14:20–32. doi:10.1038/nrmicro3552. 26499895 PMC4837114

[12] Wang J, Jia HJ 2016 Metagenome-wide association studies: fine-mining the microbiome. Nat Rev Microbiol 14:508–522. doi:10.1038/nrmicro.2016.83. 27396567

[13] Manach C, Scalbert A, Morand C, Rémésy C, Jiménez L 2004 Polyphenols: food sources and bioavailability. Am J Clin Nutr 79:727–747. 15113710 10.1093/ajcn/79.5.727

[14] Duda-Chodak A 2012 The inhibitory effect of polyphenols on human gut microbiota. J Physiol Pharmacol 63:497–503. 23211303

[15] Scalbert A, Manach C, Morand C, Rémésy C, Jiménez L 2005 Dietary polyphenols and the prevention of diseases. Crit Rev Food Sci Nutr 45:287–306. doi:10.1080/1040869059096. 16047496

[16] Vauzour D, Rodriguez-Mateos A, Corona G, Oruna-Concha MJ, Spencer JPE 2010 Polyphenols and human health: prevention of disease and mechanisms of action. Nutrients 2:1106–1131. doi:10.3390/nu2111106. 22254000 PMC3257622

[17] Manach C, Williamson G, Morand C, Scalbert A, Rémésy C 2005 Bioavailability and bioefficacy of polyphenols in humans. I. Review of 97 bioavailability studies. Am J Clin Nutr 81(1 Suppl):230–242. 10.1093/ajcn/81.1.230S 15640486

[18] Hervert-Hernández D, Goñi I 2011 Dietary polyphenols and human gut microbiota: a review. Food Rev Int 27:154–169. doi:10.1080/87559129.2010.535233.

[19] Menon R, Munjal N, Sturino JM 2015 Characterization of amygdaline-degrading *Lactobacillus* species. J Appl Microbiol 118:443–453. doi:10.1111/jam.12704. 25421573

[20] Altermann E, Russell WM, Azcarate-Peril MA, Barrangou R, Buck BL, McAuliffe O, Souther N, Dobson A, Duong T, Callanan M, Lick S, Hamrick A, Cano R, Klaenhammer TR 2005 Complete genome sequence of the probiotic lactic acid bacterium *Lactobacillus acidophilus* NCFM. Proc Natl Acad Sci U S A 102:3906–3912. doi:10.1073/pnas.0409188102. 15671160 PMC554803

[21] Sanders ME, Klaenhammer TR 2001 Invited review: the scientific basis of *Lactobacillus acidophilus* NCFM functionality as a probiotic. J Dairy Sci 84:319–331. doi:10.3168/jds.S0022-0302(01)74481-5. 11233016

[22] Pfeiler EA, Klaenhammer TR 2009 Role of transporter proteins in bile tolerance of *Lactobacillus acidophilus*. Appl Environ Microbiol 75:6013–6016. doi:10.1128/AEM.00495-09. 19633113 PMC2747850

[23] Goh YJ, Klaenhammer TR 2014 Insights into glycogen metabolism in *Lactobacillus acidophilus*: impact on carbohydrate metabolism, stress tolerance and gut retention. Microb Cell Fact 13:94. doi:10.1186/s12934-014-0094-3. 25410006 PMC4243779

[24] O’Flaherty SJ, Klaenhammer TR 2010 Functional and phenotypic characterization of a protein from *Lactobacillus acidophilus* involved in cell morphology, stress tolerance and adherence to intestinal cells. Microbiology 156:3360–3367. doi:10.1099/mic.0.043158-0. 20829293

[25] Andersen JM, Barrangou R, Abou Hachem M, Lahtinen S, Goh YJ, Svensson B, Klaenhammer TR 2011 Transcriptional and functional analysis of galactooligosaccharide uptake by lacS in *Lactobacillus acidophilus*. Proc Natl Acad Sci U S A 108:17785–17790. doi:10.1073/pnas.1114152108. 22006318 PMC3203779

[26] Andersen JM, Barrangou R, Abou Hachem M, Lahtinen SJ, Goh YJ, Svensson B, Klaenhammer TR 2012 Transcriptional analysis of prebiotic uptake and catabolism by *Lactobacillus acidophilus* NCFM. PLoS One 7:e44409. doi:10.1371/journal.pone.0044409. 23028535 PMC3446993

[27] Barrangou R, Azcarate-Peril MA, Duong T, Conners SB, Kelly RM, Klaenhammer TR 2006 Global analysis of carbohydrate utilization by *Lactobacillus acidophilus* using cDNA microarrays. Proc Natl Acad Sci U S A 103:3816–3821. doi:10.1073/pnas.0511287103. 16505367 PMC1533782

[28] Lorca GL, Barabote RD, Zlotopolski V, Tran C, Winnen B, Hvorup RN, Stonestrom AJ, Nguyen E, Huang LW, Kim DS, Saier MH 2007 Transport capabilities of eleven gram-positive bacteria: comparative genomic analyses. Biochim Biophys Acta 1768:1342–1366. doi:10.1016/j.bbamem.2007.02.007. 17490609 PMC2592090

[29] Nakai H, Baumann MJ, Petersen BO, Westphal Y, Schols H, Dilokpimol A, Abou Hachem M, Lahtinen SJ, Duus JO, Svensson B 2009 The maltodextrin transport system and metabolism in *Lactobacillus acidophilus* NCFM and production of novel α-glucosides through reverse phosphorolysis by maltose phosphorylase. FEBS J 276:7353–73655. doi:10.1111/j.1742-4658.2009.07445.x. 19919544

[30] Møller MS, Goh YJ, Rasmussen KB, Cypryk W, Celebioglu HU, Klaenhammer TR, Svensson B, Abou Hachem M 2017 An extracellular cell-attached pullulanase confers branched α-glucan utilization in human gut *Lactobacillus acidophilus*. Appl Environ Microbiol 83. doi:10.1128/AEM.00402-17. PMC5452828 28411221

[31] Yang B, Chen HQ, Song YD, Chen YQ, Zhang H, Chen W 2013 Myosine-cross-reactive antigens from four different lactic acid bacteria are fatty acid hydratases. Biotechnol Lett 35:75–81. doi:10.1007/s10529-012-1044-y. 22955678

[32] Buck BL, Altermann E, Svingerud T, Klaenhammer TR 2005 Functional analysis of putative adhesion factors in *Lactobacillus acidophilus* NCFM. Appl Environ Microbiol 71:8344–8351. doi:10.1128/AEM.71.12.8344-8351.2005. 16332821 PMC1317474

[33] Parkar SG, Stevenson DE, Skinner MA 2008 The potential influence of fruit polyphenols on colonic microflora and human gut health. Int J Food Microbiol 124:295–298. doi:10.1016/j.ijfoodmicro.2008.03.017. 18456359

[34] Lombard V, Ramulu HG, Drula E, Coutinho PM, Henrissat B 2014 The carbohydrate-active enzymes database (CAZy) in 2013. Nucleic Acids Res 42:D490–DD495. doi:10.1093/nar/gkt1178. 24270786 PMC3965031

[35] Kant R, Blom J, Palva A, Siezen RJ, de Vos WM 2011 Comparative genomics of *Lactobacillus*. Microb Biotechnol 4:323–332. doi:10.1111/j.1751-7915.2010.00215.x. 21375712 PMC3818991

[36] Goh YJ, Azcárate-Peril MA, O’Flaherty S, Durmaz E, Valence F, Jardin J, Lortal S, Klaenhammer TR 2009 Development and application of a *upp*-based counterselective gene replacement system for the study of the s-layer protein slpx of *Lactobacillus acidophilus* NCFM. Appl Environ Microbiol 75:3093–3105. doi:10.1128/AEM.02502-08. 19304841 PMC2681627

[37] Ge BY, Chen HX, Han FM, Chen Y 2007 Identification of amygdalin and its major metabolites in rat urine by LC-MS/MS. J Chromatogr B Analyt Technol Biomed Life Sci 857:281–286. doi:10.1016/j.jchromb.2007.07.036. 17720632

[38] Takos A, Lai D, Mikkelsen L, Abou Hachem M, Shelton D, Motawia MS, Olsen CE, Wang TL, Martin C, Rook F 2010 Genetic screening identifies cyanogenesis-deficient mutants of *Lotus japonicus* and reveals enzymatic specificity in hydroxynitrile glucoside metabolism. Plant Cell 22:1605–1619. doi:10.1105/tpc.109.073502. 20453117 PMC2899875

[39] Scalbert A, Andres-Lacueva C, Arita M, Kroon P, Manach C, Urpi-Sarda M, Wishart D 2011 Databases on food phytochemicals and their health-promoting effects. J Agric Food Chem 59:4331–4348. doi:10.1021/jf200591d. 21438636

[40] Landete JM, Curiel JA, Rodríguez H, de las Rivas B, Muñoz R 2014 Aryl glycosidases from *Lactobacillus plantarum* increase antioxidant activity of phenolic compounds. J Funct Foods 7:322–329. doi:10.1016/j.jff.2014.01.028.

[41] Laparra JM, Sanz Y 2010 Interactions of gut microbiota with functional food components and nutraceuticals. Pharmacol Res 61:219–225. doi:10.1016/j.phrs.2009.11.001. 19914380

[42] Possemiers S, Rabot S, Espín JC, Bruneau A, Philippe C, González-Sarrías A, Heyerick A, Tomás-Barberán FA, De Keukeleire D, Verstraete W 2008 *Eubacterium limosum* activates isoxanthohumol from hops (*Humulus lupulus* L.) into the potent phytoestrogen 8-prenylnaringenin *in vitro* and in rat intestine. J Nutr 138:1310–1316. 18567753 10.1093/jn/138.7.1310

[43] Carmody RN, Turnbaugh PJ 2014 Host-microbial interactions in the metabolism of therapeutic and diet-derived xenobiotics. J Clin Invest 124:4173–4181. doi:10.1172/JCI72335. 25105361 PMC4191041

[44] Francl AL, Thongaram T, Miller MJ 2010 The PTS transporters of *Lactobacillus gasseri* ATCC 33323. BMC Microbiol 10:77. doi:10.1186/1471-2180-10-77. 20226062 PMC2848229

[45] Deutscher J, Aké FMD, Derkaoui M, Zébré AC, Cao TN, Bouraoui H, Kentache T, Mokhtari A, Milohanic E, Joyet P 2014 The bacterial phosphoenolpyruvate:carbohydrate phosphotransferase system: regulation by protein phosphorylation and phosphorylation-dependent protein-protein interactions. Microbiol Mol Biol Rev 78:231–256. doi:10.1128/MMBR.00001-14. 24847021 PMC4054256

[46] Saier MH, Reddy VS, Tsu BV, Ahmed MS, Li C, Moreno-Hagelsieb G 2016 The Transporter Classification Database (TCDB): recent advances. Nucleic Acids Res 44:D372–D379. doi:10.1093/nar/gkv1103. 26546518 PMC4702804

[47] Manach C, Hubert J, Llorach R, Scalbert A 2009 The complex links between dietary phytochemicals and human health deciphered by metabolomics. Mol Nutr Food Res 53:1303–1315. doi:10.1002/mnfr.200800516. 19764066

[48] Dillard CJ, German JB 2000 Phytochemicals: nutraceuticals and human health. J Sci Food Agric 80:1744–1756. doi:10.1002/1097-0010(20000915)80:12<1744::AID-JSFA725>3.0.CO;2-W.

[49] Taguer M, Maurice CF 2016 The complex interplay of diet, xenobiotics, and microbial metabolism in the gut: implications for clinical outcomes. Clin Pharmacol Ther 99:588–599. doi:10.1002/cpt.366. 26950037

[50] Maurice CF, Haiser HJ, Turnbaugh PJ 2013 Xenobiotics shape the physiology and gene expression of the active human gut microbiome. Cell 152:39–50. doi:10.1016/j.cell.2012.10.052. 23332745 PMC3552296

[51] Schmid B, Kötter I, Heide L 2001 Pharmacokinetics of salicin after oral administration of a standardised willow bark extract. Eur J Clin Pharmacol 57:387–391. doi:10.1007/s002280100325. 11599656

[52] Whang WK, Park HS, Ham I, Oh M, Namkoong H, Kim HK, Hwang DW, Hur SY, Kim TE, Park YG, Kim JR, Kim JW 2005 Natural compounds, fraxin and chemicals structurally related to fraxin protect cells from oxidative stress. Exp Mol Med 37:436–446. doi:10.1038/emm.2005.54. 16264268

[53] Baur JA, Pearson KJ, Price NL, Jamieson HA, Lerin C, Kalra A, Prabhu VV, Allard JS, Lopez-Lluch G, Lewis K, Pistell PJ, Poosala S, Becker KG, Boss O, Gwinn D, Wang MY, Ramaswamy S, Fishbein KW, Spencer RG, Lakatta EG, Le Couteur D, Shaw RJ, Navas P, Puigserver P, Ingram DK, de Cabo R, Sinclair DA 2006 Resveratrol improves health and survival of mice on a high-calorie diet. Nature 444:337–342. doi:10.1038/nature05354. 17086191 PMC4990206

[54] Jang MS, Cai L, Udeani GO, Slowing KV, Thomas CF, Beecher CWW, Fong HHS, Farnsworth NR, Kinghorn AD, Mehta RG, Moon RC, Pezzuto JM 1997 Cancer chemopreventive activity of resveratrol, a natural product derived from grapes. Science 275:218–220. doi:10.1126/science.275.5297.218. 8985016

[55] Smoliga JM, Baur JA, Hausenblas HA 2011 Resveratrol and health—A comprehensive review of human clinical trials. Mol Nutr Food Res 55:1129–1141. doi:10.1002/mnfr.201100143. 21688389

[56] Setchell KDR, Clerici C 2010 Equol: history, chemistry, and formation. J Nutr 140:1355S–1362S. doi:10.3945/jn.109.119776. 20519412 PMC2884333

[57] Decroos K, Vanhemmens S, Cattoir S, Boon N, Verstraete W 2005 Isolation and characterisation of an equol-producing mixed microbial culture from a human faecal sample and its activity under gastrointestinal conditions. Arch Microbiol 183:45–55. doi:10.1007/s00203-004-0747-4. 15578160

[58] Salvetti E, Torriani S, Felis GE 2012 The genus *Lactobacillus*: A taxonomic update. Probiotics Antimicrob Proteins 4:217–226. doi:10.1007/s12602-012-9117-8. 26782181

[59] Russell WM, Klaenhammer TR 2001 Efficient system for directed integration into the *Lactobacillus acidophilus* and *Lactobacillus gasseri* chromosomes via homologous recombination. Appl Environ Microbiol 67:4361–4364. doi:10.1128/AEM.67.9.4361-4364.2001. 11526048 PMC93172

[60] Kimmel SA, Roberts RF 1998 Development of a growth medium suitable for exopolysaccharide production by *Lactobacillus delbrueckii* ssp. *bulgaricus* RR. Int J Food Microbiol 40:87–92. doi:10.1016/S0168-1605(98)00023-3. 9600614

[61] Duncan SH, Hold GL, Barcenilla A, Stewart CS, Flint HJ 2002 *Roseburia intestinalis* sp. nov., a novel saccharolytic, butyrate-producing bacterium from human faeces. Int J Syst Evol Microbiol 52:1615–1620. doi:10.1099/00207713-52-5-1615. 12361264

[62] Kearse M, Moir R, Wilson A, Stones-Havas S, Cheung M, Sturrock S, Buxton S, Cooper A, Markowitz S, Duran C, Thierer T, Ashton B, Meintjes P, Drummond A 2012 Geneious Basic: an integrated and extendable desktop software platform for the organization and analysis of sequence data. Bioinformatics 28:1647–1649. doi:10.1093/bioinformatics/bts199. 22543367 PMC3371832

[63] Goh YJ, Klaenhammer TR 2013 A functional glycogen biosynthesis pathway in *Lactobacillus acidophilus*: expression and analysis of the *glg* operon. Mol Microbiol 89:1187–1200. doi:10.1111/mmi.12338. 23879596 PMC4282360

[64] Saier MH, Reddy VS, Tamang DG, Västermark A 2014 The Transporter classification database. Nucleic Acids Res 42:D251–D258. doi:10.1093/nar/gkt1097. 24225317 PMC3964967

[65] Horton RM, Hunt HD, Ho SN, Pullen JK, Pease LR 1989 Engineering hybrid genes without the use of restriction enzymes—gene-splicing by overlap extension. Gene 77:61–68. doi:10.1016/0378-1119(89)90359-4. 2744488

[66] Wang YN, Zhao M, Ou YF, Zeng BW, Lou XY, Wang M, Zhao CJ 2016 Metabolic profile of esculin in rats by ultra high performance liquid chromatography combined with Fourier transform ion cyclotron resonance mass spectrometry. J Chromatogr B Analyt Technol Biomed Life Sci 1020:120–128. doi:10.1016/j.jchromb.2016.03.027. 27038404

[67] Kingsford CL, Ayanbule K, Salzberg SL 2007 Rapid, accurate, computational discovery of Rho-independent transcription terminators illuminates their relationship to DNA uptake. Genome Biol 8:R22. doi:10.1186/gb-2007-8-2-r22. 17313685 PMC1852404

