# Supplementary material for: Lactobacillus acidophilus Metabolizes Dietary Plant Glucosides and Externalizes Their Bioactive Phytochemicals
Source: mBio. 2017 Nov 21;8(6):e01421-17. doi: 10.1128/mBio.01421-17 (PMC5698550; doi:10.1128/mBio.01421-17)
Supplement: TABLE S1 [file mbo006173598st1.docx]

| Table S1: Plant glycosides studied in this work and their ability to support growth (*OD*_600_) of *L. acidophilus* NCFM. | | | | | | | |
| --- | --- | --- | --- | --- | --- | --- | --- |
| Compound | CAS No. | Example of a common natural source | Supplier | Purity^a^ | Growth concentration (w/v)^b^ | *OD*_600_ max^c^ | Catabolism based on MS^d^ |
| Amygdalin | 29883-15-6 | Almonds | Sigma | ≥99% | 1% | 0.3 | Yes |
| Arbutin | 497-76-7 | Pear | Sigma | ≥98% | 1% | 0.0 | No |
| Aucubin | 479-98-1 | Asterid plants | Chemfaces | ≥98% | 0.5% | 0.0 | No |
| Daidzin | 552-66-9 | Soy | AdooQ | >98% | 0.5% | 0.0 | ND |
| Esculin | 531-75-9 | Dandelion coffee | Sigma | ≥98% | 0.5% | 0.4 | Yes |
| Fraxin | 524-30-1 | Kiwi | Chemfaces | ≥98% | 0.5% | 0.4 | Yes |
| Isoquercetin | 482-35-9 | Onion | Sigma | ≥90% | 0.5% | 0.0 | No |
| Polydatin | 65914-17-2 | Grapes | Sigma | ≥95% | 0.5% | NA | Yes |
| Rutin hydrate | 153-18-4 | Tea | Sigma | ≥94% | 0.5% | 0.1 | No |
| Salicin | 138-52-3 | Willow tree | Sigma | ≥99% | 1% | 0.8 | Yes |
| Sinigrin hydrate | 3952-98-5 | Broccoli | Sigma | ≥99% | 0.5% | 0.0 | ND |
| Vanilin 4-*O*-β-glucoside | 494-08-6 | Vanilla | mybiosource.com | 100% | 0.5% | 1.3 | Yes |

^a^As provided by supplier. ^b^Concentration in single carbon source growth experiments. ^c^Maximum optical density (600 nm) corrected for growth in semi-defined medium without carbon source in 200 µl cultures in 96 microtitre plates, which corresponds to approximately 50% of the absorbance in a 1 cm cuvette. ^d^Mass spectrometry qualitative analysis of catabolism based on the depletion of the plant glycoside and/or appearance of its metabolites. ND: not detected. NA: Not applicable due to low solubility of the compound.
