# Supplementary material for: Lactobacillus acidophilus Metabolizes Dietary Plant Glucosides and Externalizes Their Bioactive Phytochemicals
Source: mBio. 2017 Nov 21;8(6):e01421-17. doi: 10.1128/mBio.01421-17 (PMC5698550; doi:10.1128/mBio.01421-17)
Supplement: TABLE S2 [file mbo006173598st2.docx]

| Table S2. Growth of *Lactobacillus* species on selected plant glucosides, cellobiose, and glucose corrected to the growth level in medium without carbohydrate. "+++" signifies *OD*_600 max_ > 0.6. "++" signifies 0.6 > *OD*_600_ _max_ > 0.3. "+" signifies 0.3 > *OD*_600_ max > 0.1. “-“ signifies *OD*_600 max_ < 0.1. | | | | | | | | |
| --- | --- | --- | --- | --- | --- | --- | --- | --- |
| Strain | Source (ref) | Genome sequence | Amygdalin | Arbutin | Esculin | Salicin | Cellobiose | Glucose |
| *L. acidophilus* LA-1 | Human^a^ |  | ++ | - | +++ | +++ | +++ | +++ |
| *L. acidophilus* NCFM | Human intestinal isolate (1) | Complete | + | - | ++ | +++ | ++ | ++ |
| *L. amylvorus* ATCC 33620, DSM 20531 | Cattle feces (2) |  | - | - | - | - | +++ | +++ |
| *L. animalis* DSM 20602 | Baboon dental plaque (3) |  | - | + | + | + | + | ++ |
| *L. crispatus* ATCC 33820, DSM 20584 | Human isolate (4) |  | - | - | ++ | +++ | - | ++ |
| *L. fermentum* ATCC 14931 | Fermented beets (5) |  | - | - | - | - | - | +++ |
| *L. gasseri* ATCC 33323 | Human isolate (6) |  | - | - | ++ | ++ | ++ | +++ |
| *L. helveticus* CNRZ32 | Industrial cheese starter culture (7) |  | + | - | - | - | + | ++ |
| *L. intestinalis* Th4, ATCC 49335, DSM 6629 | Rat intestine (8) | Scaffold | - | - | - | - | - | ++ |
| *L. jensenii* ATCC 25258, 62G, DSM 20557 | Human vaginal discharge (9) | Scaffold | + | + | ++ | ++ | ++ | ++ |
| *L. johnsonii* ATCC 33200 | Human blood (10) | Contig | - | + | - | + | + | +++ |
| *L. plantarum* sp. *plantarum* ATCC 14917, LA70 | Pickled cabbage, human microbiome project (5) | Scaffold | +++ | +++ | ++ | +++ | +++ | +++ |
| *L. reuteri* (ATCC 23272, DSM 20016) | Human feces (11) |  | - | - | - | - | - | ++ |
| *L. rhamnosus* GG (ATCC 53103) | Human feces (12) |  | +++ | +++ | ++ | +++ | +++ | +++ |
| ^a^From Nestec (Lausanne, Switzerland) collection. | | | | | | | | |

**Table S2 references**

1. **Altermann E, Russell WM, Azcarate-Peril MA, Barrangou R, Buck BL, McAuliffe O, Souther N, Dobson A, Duong T, Callanan M, Lick S, Hamrick A, Cano R, Klaenhammer TR.** 2005. Complete genome sequence of the probiotic lactic acid bacterium *Lactobacillus acidophilus* NCFM. Proc Natl Acad Sci U S A **102:**3906–3912.

2. **Nakamura LK.** 1981. *Lactobacillus amylovorus*, a new starch-hydrolyzing species from cattle waste-corn fermentations. Int J Syst Bacteriol **31:**56-63.

3. **Dent VE, Williams RAD.** 1982. Lactobacillus animalis sp. nov., a new species of *Lactobacillus* from the alimentary canal of animals. Zentralblatt Fur Bakteriologie Mikrobiologie Und Hygiene I Abteilung Originale C-Allgemeine Angewandte Und Okologische Mikrobiologie **3:**377-386.

4. **Cato EP, Moore WEC, Johnson JL.** 1983. Synonymy of strains of *Lactobacillus acidophilus* group A2 (Johnson et al 1980) with the type strain of *Lactobacillus crispatus* (Brygoo and Aladame 1953) Moore and Holdeman 1970 International Journal of Systematic Bacteriology **33:**426-428.

5. **Skerman VBD, McGowan V, Sneath PHA.** 1980. Approved lists of bacterial names. International Journal of Systematic Bacteriology **30:**225-420.

6. **Lauer E, Kandler O.** 1980. *Lactobacillus gasseri* sp. nov., a new species of the subgenus Thermobacterium Zentralblatt Fur Bakteriologie Mikrobiologie Und Hygiene I Abteilung Originale C-Allgemeine Angewandte Und Okologische Mikrobiologie **1:**75-78.

7. **Broadbent JR, Hughes JE, Welker DL, Tompkins TA, Steele JL.** 2013. Complete Genome Sequence for *Lactobacillus helveticus* CNRZ 32, an IndustrialCheese Starter and Cheese Flavor Adjunct.Complete Genome Sequence for Lactobacillus helveticus CNRZ 32, an IndustrialCheese Starter and Cheese Flavor Adjunct. Genome Announcements **1:**1-2.

8. **Fujisawa T, Itoh K, Benno Y, Mitsuoka T.** 1990. *Lactobacillus intestinalis* (ex Hemme 1974) sp. nov., nom. rev., isolated from the intestines of mice and rats. International Journal of Systematic Bacteriology **40:**302-304.

9. **Gasser F, Mandel M, Rogosa M.** 1970. *Lactobacillus jensenii* sp. nov., a new representative of subgenus Thermobacterium. Journal of General Microbiology **62:**219-&amp;.

10. **Fujisawa T, Benno Y, Yaeshima T, Mitsuoka T.** 1992. Taxonomic study of the *Lactobacillus acidophilus* group, with recognition of *Lactobacillus gallinarum* sp. nov. and *Lactobacillus johnsonii* sp. nov. and synonymy of *Lactobacillus acidophilus*  group A3 (Johnson et al 1980) with the type strain of  *Lactobacillus amylovorus* (Nakamura 1981). International Journal of Systematic Bacteriology **42:**487-491.

11. **Kandler O, Stetter KO, Kohl R.** 1980. *Lactobacillus reuteri* sp. nov., a new species of heterofermentative lactobacilli. Zentralblatt Fur Bakteriologie Mikrobiologie Und Hygiene I Abteilung Originale C-Allgemeine Angewandte Und Okologische Mikrobiologie **1:**264-269.

12. **Silva M, Jacobus NV, Deneke C, Gorbach SL.** 1987. Antimicrobial substance from a human *Lactobacillus* strain. Antimicrobial Agents and Chemotherapy **31:**1231-1233.
