# Supplementary material for: Lactobacillus acidophilus Metabolizes Dietary Plant Glucosides and Externalizes Their Bioactive Phytochemicals
Source: mBio. 2017 Nov 21;8(6):e01421-17. doi: 10.1128/mBio.01421-17 (PMC5698550; doi:10.1128/mBio.01421-17)
Supplement: TABLE S4 [file mbo006173598st4.docx]

| Table S4. Conservation of plant glycoside utilization gene loci identified in this work (annotated by their locus tags and accession numbers) in *Lactobacillus acidophilus* strains in the NCBI organism database. The table shows the amino acid identities and the sequence coverage if it is less than 100% of the protein in *L. acidophilus* NCFM. | | | | | | | | | | | | | |
| --- | --- | --- | --- | --- | --- | --- | --- | --- | --- | --- | --- | --- | --- |
| Strain | BioProject | Size (Mb) | Scaffolds | Genes | Proteins | ID as compared to *L. acidophilus* NCFM genes (loci and accession no.) | | | | | | | |
|  |  |  |  |  |  | LBA0225 | LBA0226 | LBA0227 | LBA0228 | LBA0724 | LBA0725 | LBA0726 | LBA0728 |
|  |  |  |  |  |  | AAV42120.1 | AAV42121.1 | AAV42122.1 | AAV42123.1 | AAV42595.1 | AAV42596.1 | AAV42597.1 | AAV42598.1 |
| NCFM | PRJNA82 | 1.99 | 1 | 1927 | 1832 | 100% | 100% | 100% | 100% | 100% | 100% | 100% | 100% |
| La-14 | PRJNA196176 | 1.99 | 1 | 1948 | 1835 | 100% | 100% | 100% | 100% | 100%  (Cover: 99%) | 99% | 100% | 100% |
| FSI4 | PRJNA271341 | 1.99 | 1 | 1948 | 1845 | 100% | 100% | 100% | 100% | 100%  (Cover: 99%) | 99% | 100% | 100% |
| ATCC 4796 | PRJNA31477 | 2.02 | 38 | 1957 | 1802 | 100%  (Cover: 71%) | 100% | 100% | 99% | 100%  (Cover: 99%) | 99% | 100% | 99% |
| CIP 76.13 | PRJEB1532 | 1.95 | 34 | 1935 | 1779 | 100% | 100% | 100% | 100% | 100%  (Cover: 99%) | 99% | 100% | 100% |
| DSM 9126 | PRJEB1839 | 1.99 | 27 | 1944 | 1827 | 100% | 100% | 99% | 100% | 100%  (Cover: 99%) | 99% | 100% | 100% |
| CIRM-BIA 445 | PRJEB1531 | 2.00 | 22 | 1937 | 1819 | 100% | 100% | 100% | 100% | 99% | 99% | 100% | 100% |
| DSM 20079 | PRJNA222257 | 1.95 | 30 | 1913 | 1787 | 100% | 100% | 100% | 100% | 100%  (Cover: 99%) | 99% | 100% | 100% |
| DSM 20242 | PRJEB1533 | 2.05 | 21 | 1987 | 1865 | 100% | 100% | 100% | 100% | 99% | 99% | 100% | 100% |
| CIRM-BIA 442 | PRJEB1530 | 1.99 | 19 | 1947 | 1841 | 100% | 100% | 100% | 100% | 100%  (Cover: 99%) | 99% | 100% | 100% |
| ATCC 4356 | PRJNA263693 | 1.96 | 20 | 1914 | 1780 | 100% | 100% | 100% | 100% | 100%  (Cover: 99%) | 99% | 100% | 100% |
| WG-LB-IV | PRJNA317797 | 1.95 | 74 | 1944 | 1815 | 100% | 100% | 100% | 100% | 100% | 99% | 100% | 100% |
