# Supplementary material for: Lactobacillus acidophilus Metabolizes Dietary Plant Glucosides and Externalizes Their Bioactive Phytochemicals
Source: mBio. 2017 Nov 21;8(6):e01421-17. doi: 10.1128/mBio.01421-17 (PMC5698550; doi:10.1128/mBio.01421-17)
Supplement: TABLE S5 [file mbo006173598st5.docx]

| Table S5. Strains used and constructed for gene deletion mutants in *L. acidophilus* NCFM. | | |
| --- | --- | --- |
| Strain | Source, genotype or characteristics/description | Reference |
| *Escherichia coli* EC101 | RepA^+^ JM101; Km^r^; *repA* gene from integration of pWV01 in the chromosome; cloning host for pORI-based plasmids+ | (1) |
| *Lactobacillus acidophilus* |  |  |
| NCFM | Human intestinal isolate | (2) |
| NCK1909 (*Δupp* ) | NCFM with a 0.3 kb in-frame deletion within the *upp* gene (LBA0770); background/parent strain for NCFM deletion mutants | (3) |
| NCK1910 | NCK1909 harboring the plasmid pTRK669 (4) | (3) |
| NCK2416 (*ΔLBA0225*) | NCK1909 with a 1.3 kb in-frame deletion within LBA0225 | This study |
| NCK2418 (*ΔLBA0227*) | NCK1909 with a 1.2 kb in-frame deletion within LBA0227 | This study |
| NCK2422 (*ΔLBA0725*) | NCK1909 with a 1.9 kb in-frame deletion within LBA0725 | This study |
| NCK2424 (*ΔLBA0726*) | NCK1909 with a 1.3 kb in-frame deletion within LBA0726 | This study |
| NCK2426 (*ΔLBA0225ΔLBA0726*) | NCK2416 with a 1.3 kb in-frame deletion within LBA0726 | This study |

Supplemental material references

1. **Law J, Buist G, Haandrikman A, Kok J, Venema G, Leenhouts K.** 1995. A  system to generate chromosomal mutations in *Lactococcus lactis* which allows fast analysis of targeted genes. Journal of Bacteriology **177:**7011-7018.

2. **Sanders ME, Klaenhammer TR.** 2001. The scientific basis of *Lactobacillus acidophilus* NCFM functionality as a probiotic. Journal of Dairy Science **84:**319-331.

3. **Goh YJ, Azcarate-Peril MA, O'Flaherty S, Durmaz E, Valence F, Jardin J, Lortal S, Klaenhammer TR.** 2009. Development and application of a upp-based counterselective gene replacement system for the study of the s-layer protein slpx of *Lactobacillus acidophilus* NCFM. Appl Environ Microbiol **75:**3093-3105.

4. **Russell WM, Klaenhammer TR.** 2001. Efficient system for directed integration into the *Lactobacillus acidophilus* and *Lactobacillus gasseri* chromosomes via homologous recombination. Appl Environ Microbiol **67:**4361-4364.
