# Supplementary material for: Lactobacillus acidophilus Metabolizes Dietary Plant Glucosides and Externalizes Their Bioactive Phytochemicals
Source: mBio. 2017 Nov 21;8(6):e01421-17. doi: 10.1128/mBio.01421-17 (PMC5698550; doi:10.1128/mBio.01421-17)
Supplement: TABLE S6 [file mbo006173598st6.docx]

| Table S6. Plant glycosides and their metabolites in *L. acidophilus* NCFM culture supernatants as analyzed by UHPLC-qTOF-MS. The starting plant glycoside substrates, which were identified in the cultures before inoculation, are in bold and underlined. The aglycones are in bold and the metabolite analyses were carried out from the 24h culture supernatant samples. | | | | | | | | |
| --- | --- | --- | --- | --- | --- | --- | --- | --- |
| Sample^a^ | Compound ID | Formula | UV Rt^b^ (min) | MS Rt^c^ (min) | Primary ion mode | Calc.  *m/z* | Identified *m/z* | ppm |
| Amy | **Amygdalin^d^** | C_20_H_27_NO_11_ | 3.45 | 3.535 | [M+HCOO]^-^ | 502.1566 | 502.1569 | 0.3 |
| Amy | Benzaldehyde^d^ | C_7_H_6_O | 6.04 | ND^e^ | - | - | - | - |
| Amy | Prunasin | C_14_H_17_NO_6_ | ND | 4.163 | [M+HCOO]^-^ | 340.1038 | 340.1032 | 2.01 |
| Arb | **Arbutin^d^** | C_12_H_16_O_7_ | 1.98 | 2.055 | [M+HCOO]^-^ | 317.0878 | 317.088 | 0.17 |
| Auc | **Aucubin** | C_15_H_22_O_9_ | ND | 2.00^f^ | [M+HCOO]^-^ | 391.1246 | 391.1252 | 1.28 |
| Esc | **Esculin^d^** | C_15_H_16_O_9_ | 2.94 | 3.008 | [M-H]^-^ | 339.0722 | 339.0726 | 1.02 |
| Esc | **Esculetin^d^** | C_9_H_6_O_4_ | 3.59 | 3.666 | [M-H]^-^ | 177.0193 | 177.0196 | 1.25 |
| Esc | Scopeletin | C_10_H_8_O_4_ | 4.86 | 4.719 | [M-H]^-^ | 191.0350 | 191.0345 | 3.28 |
| Fra | **Fraxin** | C_16_H_18_O_10_ | 3.47 | 3.525 | [M-H]^-^ | 369.0827 | 369.083 | 0.27 |
| Fra | **Fraxetin** | C_10_H_8_O_5_ | 4.04 | 4.120 | [M-H]^-^ | 207.0299 | 207.03 | 0.06 |
| Fra | Esculin^d^ | C_15_H_16_O_9_ | 2.96 | 3.013 | [M-H]^-^ | 339.0722 | 339.0719 | 1.19 |
| Fra | Esculetin^d^ | C_9_H_6_O_4_ | 3.62 | 3.678 | [M-H]^-^ | 177.0193 | 177.019 | 2.79 |
| Fra | Scopeletin | C_10_H_8_O_4_ | 4.86 | 4.869 | [M-H]^-^ | 191.0350 | 191.0352 | 0.77 |
| IQ | **Isoquercetin** | C_21_H_20_O_12_ | 4.31 | 4.382 | [M-H]^-^ | 463.0882 | 463.0884 | 0.14 |
| PD | **Polydatin** | C_20_H_22_O_8_ | 4.37 | 4.434 | [M+HCOO]^-^ | 435.1297 | 435.1299 | 0.46 |
| PD | **Resveratrol** | C_14_H_12_O_3_ | 5.69 | 5.764 | [M-H]^-^ | 227.0714 | 227.0716 | 0.13 |
| Rut | **Rutin** | C_27_H_30_O_16_ | 4.08 | 4.15 | [M-H]^-^ | 609.1461 | 609.1467 | 0.76 |
| Sal | **Salicin^d^** | C_13_H_18_O_7_ | 2.79 | 2.865 | [M+HCOO]^-^ | 331.1035 | 331.1032 | 1.17 |
| Sal | **Salicyl alcohol^d^** | C_7_H_8_O_2_ | 3.52 | 3.592 | [M-H]^-^ | 123.0452 | 123.0458 | 0.88 |
| Van | **Vanillin 4-O-β-Glc*p*** | C_14_H_18_O_8_ | 3.12 | 3.178 | [M+HCOO]^-^ | 359.0984 | 359.0984 | 0.86 |
| Van | **Vanillin** | C_8_H_8_O_3_ | 4.61 | 4.683 | [M-H]^-^ | 151.0401 | 151.0401 | 0.34 |
| ^a^Supernatant of *L. acidophilus* NCFM growing on amygdalin (Amy), arbutin (Arb), aucubin (Auc), esculin (Esc), fraxin (Fra), polydatin (PD), isoquercetin (IQ), rutin (Rut), salicin (Sal), or vanillin 4-*O-*β-glucoside (Van). ^b^Retention time measured in the UV detector. ^c^Retention time in the MS detector. ^d^Confirmed by comparison with standard compounds. ^e^Not detected. ^f^Compound eluted in several peaks, where the predominant one is noted. | | | | | | | | |
