# Supplementary material for: Lactobacillus acidophilus Metabolizes Dietary Plant Glucosides and Externalizes Their Bioactive Phytochemicals
Source: mBio. 2017 Nov 21;8(6):e01421-17. doi: 10.1128/mBio.01421-17 (PMC5698550; doi:10.1128/mBio.01421-17)
Supplement: FIG S1 [file mbo006173598sf1.pdf]

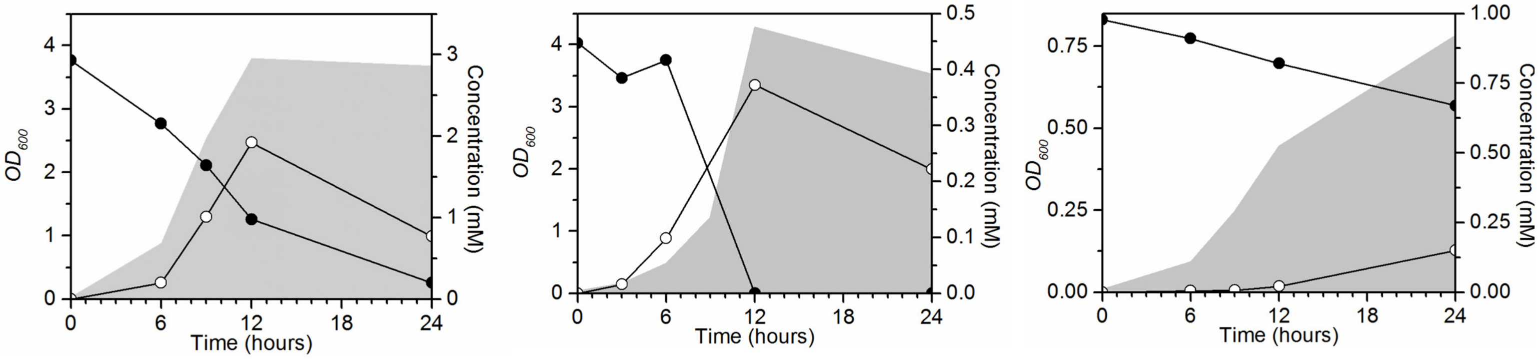

Fig. S1. Time-resolved metabolite analysis of salicin (left panel), esculin (middle panel), and amygdalin (right panel) in L. acidophilus NCFM culture supernatants as analyzed by UHPLC-qTOF-MS. The growth (measured as OD600 and shown as light gray background) is shown together with the concentrations of plant glycosides (full circles) and their or main metabolites (hollow circle).
