# Supplementary material for: Lactobacillus acidophilus Metabolizes Dietary Plant Glucosides and Externalizes Their Bioactive Phytochemicals
Source: mBio. 2017 Nov 21;8(6):e01421-17. doi: 10.1128/mBio.01421-17 (PMC5698550; doi:10.1128/mBio.01421-17)
Supplement: FIG S2 [file mbo006173598sf2.pdf]

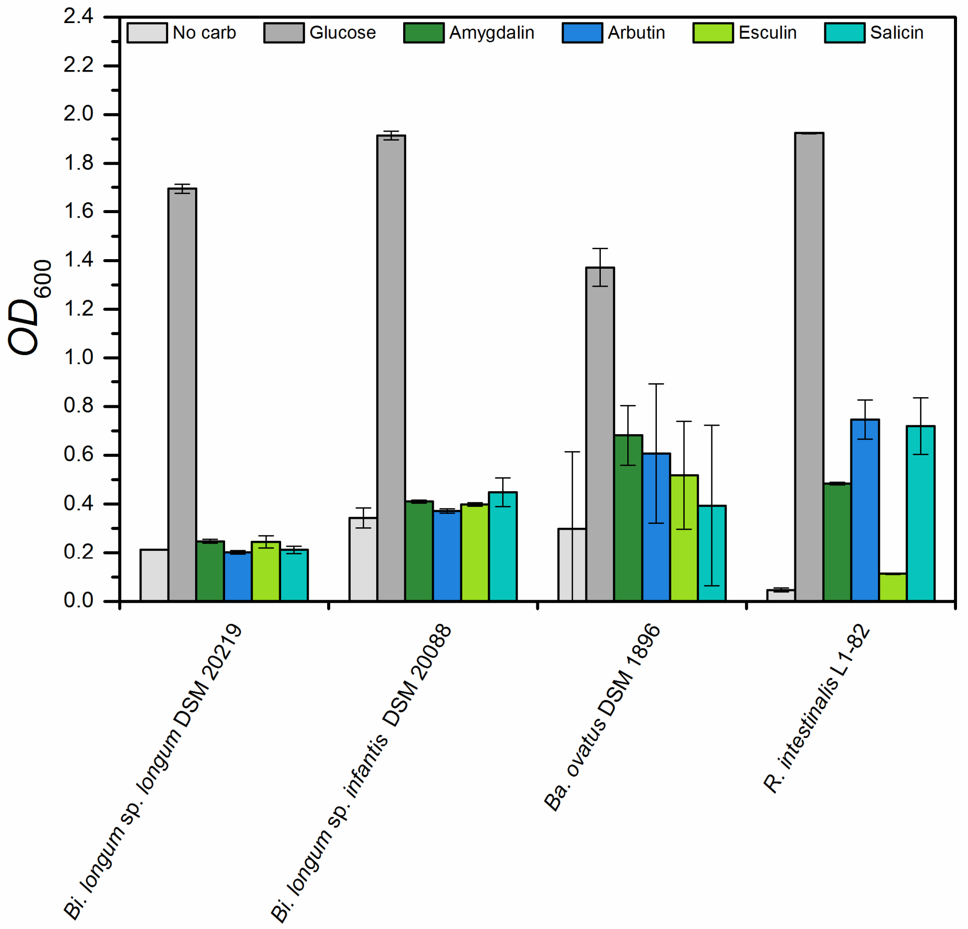

Fig. S2. Growth of common human gut microbiota commensals from the *Bifidobacterium* (*Bi*), *Bacteroides* (*Ba*) and *Roseburia* (R) genera on plant glycosides. The strains were grown in modified MRS medium except *Roseburia intestinalis,* which was grown in YCFA medium (1). The media were supplemented with 0.5% (w/v) of respective substrate and a control without any supplementation was included (no carb). Growth as OD_{600} was measured after 24h growth. The growth data is reported as means of biological duplicates with standard deviations.
