# Supplementary material for: Lactobacillus acidophilus Metabolizes Dietary Plant Glucosides and Externalizes Their Bioactive Phytochemicals
Source: mBio. 2017 Nov 21;8(6):e01421-17. doi: 10.1128/mBio.01421-17 (PMC5698550; doi:10.1128/mBio.01421-17)
Supplement: TABLE S7 [file mbo006173598st7.docx]

| Table S7. Primers used in this study. | | |
| --- | --- | --- |
| Name | Sequence | Primer usage |
| LBA0225A | GTAATAGGATCCCAACCATAGTTCATATCAAGTGGAA | PCR |
| LBA0225B | AAGTTGATGAGCGGCAACAG | PCR |
| LBA0225C | CTGTTGCCGCTCATCAACTTCAAAATGTGATTAAAACAAATGGCC | PCR |
| LBA0225D | TTAGTAGAGCTCGACTTGCATGCACCACAAAT | PCR |
| LBA0225*up* | TGCTCAAAACGCACATGTTTCA | Seq/Control |
| LBA0225*down* | ACTCGTGCTCGTGAACCAAT | Seq/Control |
| LBA0225*mid* | GAACACTATGTTCCATCTTAGGAAAA | Seq/Control |
| LBA0227A | GTAATAGGATCCGGTAGTATTAGCTAATTTAGGAACA | PCR |
| LBA0227B | TAATGCAACGATTGGTCTTG | PCR |
| LBA0227C | CAAGACCAATCGTTGCATTACTCTACAAGCAGGAACAACA | PCR |
| LBA0227D | TTAGTAGAATTCAATCCTTATTTCCGGTAGCT | PCR |
| LBA0227*up* | GTTGTTAACGAATCTGTTGATCA | Seq/Control |
| LBA0227*down* | ATCGTTTAAAAATTGCCATTGC | Seq/Control |
| LBA0227*mid* | TCAACGGTAGATAATGACGA | Seq/Control |
| LBA0227.F | AGATGCAGAACACGGTGGTC | RT-qPCR |
| LBA0227.R | GTCCAATAGTCATTCCTGCACC | RT-qPCR |
| LBA0383.F | TACTCAAAGAAGGCTTACG' | RT-qPCR |
| LBA0383.R | ATTAACTACGGCTTGAACC | RT-qPCR |
| LBA0574.F | GGCAACCGTTGTGATGGTTATC | RT-qPCR |
| LBA0574.R | ACCTTGCAAAGTTTCTTGGGC | RT-qPCR |
| LBA0606.F | TACCGGTCTTCACCACTTGG | RT-qPCR |
| LBA0606.R | GCTGCGTATTCTGCAAGGTG | RT-qPCR |
| LBA0725A | GTAATAGGATCCTCACATTGATTTTGCCGTTACT | PCR |
| LBA0725B | TCTTTGCCACCAACATCTTT | PCR |
| LBA0725C | AAAGATGTTGGTGGCAAAGAACATCAGTTAATGGACAAGTGC | PCR |
| LBA0725D | TTAGTAGAGCTCTCTAGCATCATTACGGCTGT | PCR |
| LBA0725*up* | CAGGTTAAAGAGTTTAAATCACAAACA | Seq/Control |
| LBA0725*down* | CACGAGCACTTGCAACAAAT | Seq/Control |
| LBA0725*mid* | TGAACTGGACATTAGATTCAGACGA | Seq/Control |
| LBA0725.F | ATCTTCGGTGTTCACTGGGG | RT-qPCR |
| LBA0725.R | AAACAACCCCGATTTGTGCG | RT-qPCR |
| LBA0726A | GTAATAGGATCCAAGTCAGTAGATGCAAAATATGA | PCR |
| LBA0726B | GTAGGCACCTTCAATTTGAT | PCR |
| LBA0726C | ATCAAATTGAAGGTGCCTACTCACTTAAGAGACTTCCTAAGGA | PCR |
| LBA0726D | TTAGTAGAATTCAGTCCGCTTGTCATCATAGT | PCR |
| LBA0726*up* | AAGGGGGTTCAATGACTCAAA | Seq/Control |
| LBA0726*down* | GCTTCATACAAAAATTCAGATTTGACA | Seq/Control |
| LBA0726*mid* | TTGTTAAAGGTGAAGTAAAGGTAGG | Seq/Control |
| LBA1611.F | TGCTTGGTCCTTAGCTGGTG | RT-qPCR |
| LBA1611.R | CAATGCCGCAGTAACCGAAG | RT-qPCR |
| LBA1812.F | TCCCAGATACCTGAAACGCC | RT-qPCR |
| LBA1812.R | AAATGAAGTTTGGCCAGGCG | RT-qPCR |
| LBA1872.F | CCGCGTTGCAGATACATCAAC | RT-qPCR |
